# Bisindole Compounds—Synthesis and Medicinal Properties

**DOI:** 10.3390/antibiotics13121212

**Published:** 2024-12-13

**Authors:** Maria Marinescu

**Affiliations:** Department of Organic Chemistry, Biochemistry and Catalysis, Faculty of Chemistry, University of Bucharest, Soseaua Panduri, 030018 Bucharest, Romania; maria.marinescu@chimie.unibuc.ro

**Keywords:** indole, pharmacophore, antimicrobial, antiviral, pharmaceutical properties

## Abstract

The indole nucleus stands out as a pharmacophore, among other aromatic heterocyclic compounds with remarkable therapeutic properties, such as benzimidazole, pyridine, quinoline, benzothiazole, and others. Moreover, a series of recent studies refer to strategies for the synthesis of bisindole derivatives, with various medicinal properties, such as antimicrobial, antiviral, anticancer, anti-Alzheimer, anti-inflammatory, antioxidant, antidiabetic, etc. Also, a series of natural bisindole compounds are mentioned in the literature for their various biological properties and as a starting point in the synthesis of other related bisindoles. Drawing from these data, we have proposed in this review to provide an overview of the synthesis techniques and medicinal qualities of the bisindolic compounds that have been mentioned in recent literature from 2010 to 2024 as well as their numerous uses in the chemistry of materials, nanomaterials, dyes, polymers, and corrosion inhibitors.

## 1. Introduction

**Figure 1:**
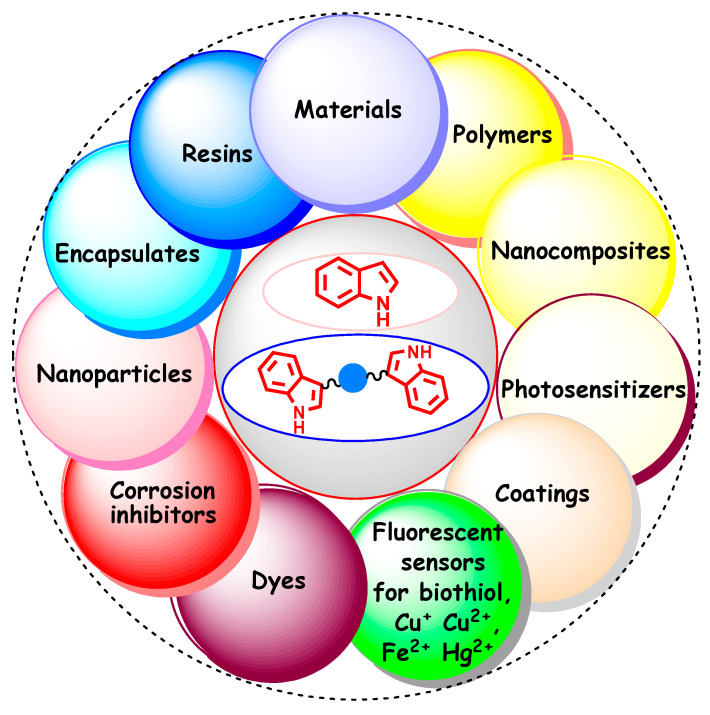
Material applications of indole and bisindole compounds with medicinal properties reported in the literature [24,25,26,27,28,29,30,31,32,33,34,35,36,37,38].

The indole ring holds a preferred position among aromatic heterocyclic compounds that contain nitrogen [1,2,3]. This is due to the fact that both natural [4,5,6,7] and synthetic compounds [8,9,10,11] are becoming more and more well known and reported in the literature for their medicinal properties [2,12,13], both as compounds with a single indole nucleus [14,15] or other heterocyclic nuclei [16,17,18]; with organic functional groups [19,20,21,22,23]; and as materials [24,25], nanoparticles [26,27,28], encapsulates [29,30], photosensitizers [31,32], polymers [33,34], resins [35,36], coatings [37], or nanocomposites [38] (Figure 1), which define their scope of use and place them at the top of the heterocycles with a wide range of biological uses and applications. Simple natural compounds, such as Tryptophan—an essential amino acid; Indole-3-carbinol—found in cruciferous vegetables with anti-carcinogenic, antioxidant, and anti-atherogenic effects; Serotonin—a tissue hormone and neurotransmitter widespread in nature and found in plantains, pineapples, banana, kiwis, plums, tomatoes, and cocoa; Melatonin—a hormone produced from Serotonin in the pineal gland with an important role in coordinating the circadian rhythmic processes in the body; Indole-3-acetic acid; and the most common naturally occurring phytohormones, are among the most famous and important indoles. The recent literature (2010–2024) reviewed in this article mentions new indolic structures, synthesized in the laboratory, with remarkable therapeutic properties.

Among the natural compounds, a series of indole alkaloids stand out; these come from vegetable sources (plants) [39,40,41], marine sources [42,43], or microorganisms [44] and are metabolites of particular biological importance [45,46]. Indole natural compounds possess a variety of pharmacological activities, including anticancer, antibacterial, antiviral, antimalarial, antifungal, anti-inflammatory, antidepressant, antiplatelet, analgesic, hypotensive, anticholinesterase, antidiarrheal, spasmolytic, antileishmanial, lipid-lowering, and antidiabetic activities [40,41,47]. Also, synthetic indoles show a variety of therapeutic activities, such as antitumor [48,49], antidiabetic [50], antihypertensive [13], antimicrobial [51,52,53,54,55,56], antiviral [57,58,59], antimalarial [60] antiparasitic [61], tuberculostatic [62,63], anticonvulsant [64], calcium channel blocking [65,66], antidepressant [67,68,69], anti-Alzheimer [70,71,72], efflux pump inhibiting [73], antioxidant [74], anti-inflammatory and analgesic [75,76], AchE and GTS inhibiting [77], and metallo-β-lactamase inhibiting [78]. Last but not least, in a series of studies, new indole compounds were mentioned as anti-SARS-CoV-2 agents, with particular importance in the context of the recent pandemic, which has further stimulated the study of synthesis methods, antiviral properties, and structure–property relations [79,80,81].

In addition, the interest of the scientific community in bisindoles has further increased due to their increased applications compared to indoles, being also reported as fluorescent sensors for biothiol, Cu^+^, Cu^2+^, Fe^2+^, and Hg^2+^ [82]; dyes [83]; and corrosion inhibitors [84] (Figure 1).

**Figure 2:**
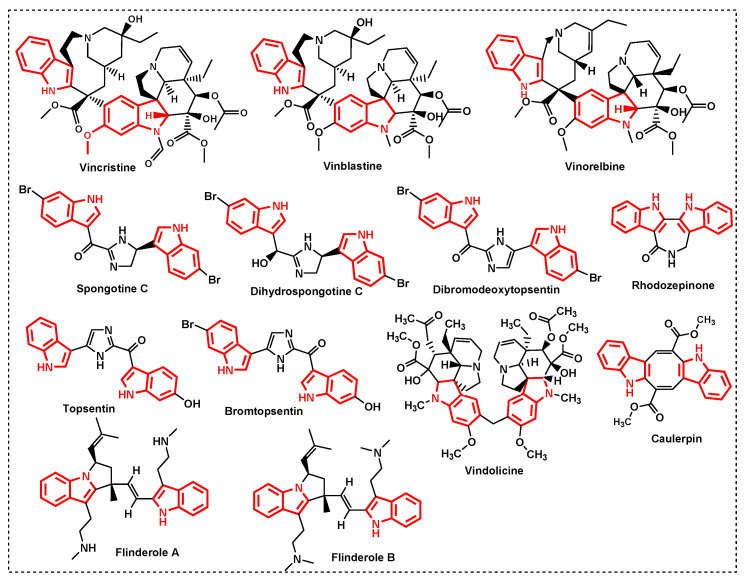
Natural bisindolic compounds with various therapeutic properties.

The present study of bisindolic compounds started from the knowledge of some natural bisindolic compounds with remarkable therapeutic properties, such as anticancer agents (vincristine, vinblastine, vinorelbine), antimicrobials (spongotin C, dihydrospongotin C, dibromodeoxytopsentin, Rhodozepinone), antimalarials (Flinderole A, Flinderole B), antivirals (topsentin, bromotopsentoin), antidiabetics (Vindolicine), anti-inflammatories (caulerpin, bromotopsentin), analgesics (caulerpin) (Figure 2), and so on [6,40,43,85].

**Figure 3:**
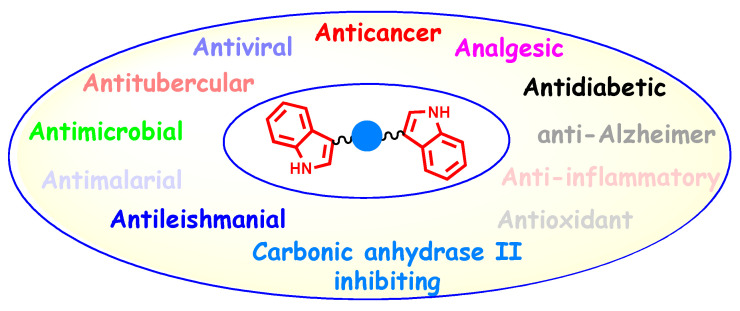
Medicinal properties of bisindolic compounds.

In this study, the synthesis techniques of bisindoles published in the literature are referred to, along with their varied therapeutic properties, as a continuation of the research reported on the synthesis and medicinal qualities of indoles. The bisindolic compounds analyzed in this article possess remarkable medicinal properties, being mentioned as anticancer, antimicrobial, antitubercular, antimalarial, antileishmanial, antiviral, analgesic, anti-Alzheimer, antidiabetic, carbonic anhydrase II inhibiting, anti-inflammatory, and antioxidant agents (Figure 3). This review aims to present synthetic bisindoles and not the natural ones.

The abundance of bisindolic compounds with exceptional antibacterial and antiviral capabilities, including novel anti-SARS-CoV-2 medicines, is the primary driving force behind the selection of this review topic. In light of the recent pandemic, the need to find novel anti-SARS-CoV-2 drugs has grown, prompting research into the synthesis processes, antimicrobial characteristics, structure–property relationships, and biological activities of bisindole compounds [79,80,81]. Also, new bisindoles with antitumor properties, which are necessary for the treatment of cancer, as well as bisindole derivatives that possess other therapeutic properties, are discussed in this review.

The database search strategy for this review included searching a number of websites, including ACS Publications, PubMed, MDPI, Science Direct, Springer, The Royal Society of Chemistry, and Taylor & Francis, using keywords, such as indole, bisindole, multicomponent reaction, indole hybrids, antimicrobial, antiviral, antitumoral, or therapeutic properties. Scientific studies over the last fifteen years were chosen based on the innovations of bisindolic compounds and their potential medical uses.

Given the evolution of synthesis methods, we begin this discussion by going over the various ways bisindoles can be synthesized before examining how they can be utilized to produce derivatives with therapeutic properties.

## 2. Synthetic Strategies for Bisindolic Compounds

### 2.1. Synthesis of Bisindolic Compounds by Electrophilic Substitution at 3-Position of Indole with Various Aryl/Heteroaryl Aldehydes

**Scheme 1:**
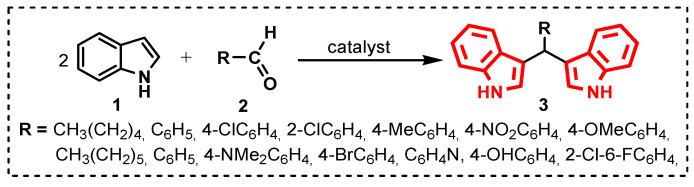
Synthesis of bis(indolyl)methanes **3** using different catalysts.

The synthesis of bisindolic compounds **3** from indole **1** and aromatic aldehydes **2**, in the presence of catalysts and various reaction conditions, is one of the main methods of their synthesis. Scheme 1 lists a few of the aldehydes that could be utilized. A number of catalysts for this reaction that produce adequate to outstanding yields are mentioned in the current literature, as can be seen in Table 1 [86,87,88,89,90,91,92,93,94,95,96,97,98,99,100,101,102,103].

### 2.2. Synthesis of Bisindole Compounds by Alkylation of Indoles with Alcohols Under Air

**Scheme 2:**
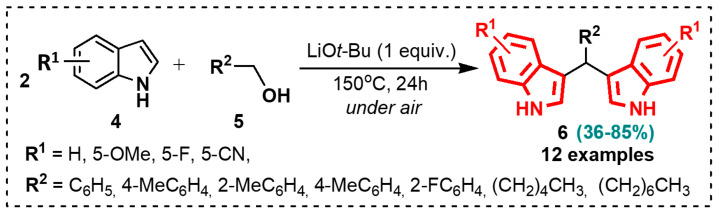
Synthesis of bis(indolyl)methanes **6** by LiO*t*-Bu-promoted alkylation of indoles **4**.

Nguyen et al. (2024) developed a convenient method for the synthesis of bis(3-indolyl)methane derivatives **6** by LiO*t*-Bu-promoted alkylation of indoles **4** with alcohols **5** under solvent-free conditions in air (Scheme 2). They also successfully used unactivated aliphatic alcohols, such as *n*-hexanol and *n*-octanol, as indole alkylation reagents, with good yields of 75% and 60% of the desired products, respectively [104].

### 2.3. Synthesis of Bis(indolyl)methane Phosphonates by Coupling of Indoles with Acyl Phosphonates

**Scheme 3:**
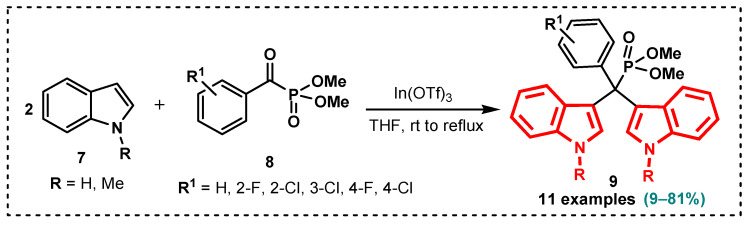
Synthesis of bis(indolyl)methane phosphonates **9** using In(OTf)_3_ as catalyst.

Dasbasi et al. (2011) described an efficient, high selectivity, and high catalytic activity protocol for synthesis of bis(indolyl)methane phosphonates **9** from N-methylindoles **7** and acyl phosphonates **8** using 10 mol % of In(OTf)_3_ as a catalyst (Scheme 3). The most effective Lewis acid for this conversion was determined to be In(OTf)_3_ (10 mol%), while other Lewis acids, including Bi(OTf)_3_, Cu(OTf)_2_, InCl_3_, InBr_3_, InI_3_, YCl_3_, Y(OTf)_3_, Sc(OTf)_3_, and BiCl_3_, were also examined [105].

### 2.4. Regioselective Synthesis of 3,3-Bis(indolyl)propanoic Acid Derivatives by Iron(III)-Catalyzed Hydroarylation Reaction of Electron-Deficient Propynoic Acid Derivatives with Indoles

**Scheme 4:**
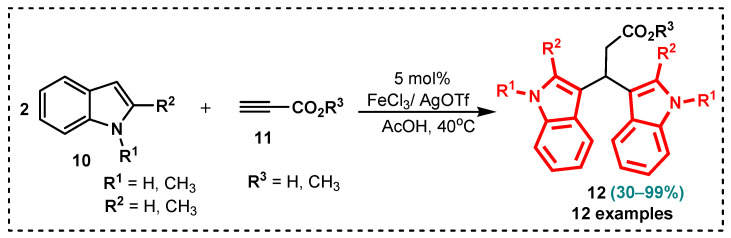
Synthesis of 3,3-bis(indol-3-yl)propanoates **12** FeCl_3_/AgOTf as catalyst.

Kutubi and Kitamura (2011) found that the catalytic system of FeCl_3_/AgOTf in acetic acid media can be efficiently used for the double hydroarylation reaction of electron-deficient propynoic acid derivatives **11** with indoles **10** (Scheme 4). The hydroarylation reaction occurs regioselectively at the 3-position of indoles **10** to give 3,3′-bis(indol-3-yl) propanoates **12** in high yields [106].

### 2.5. Synthesis of Bis(indolyl)methanes by Electrophilic Substitution in the 3-Position of Indoles with Ketones

**Scheme 5:**
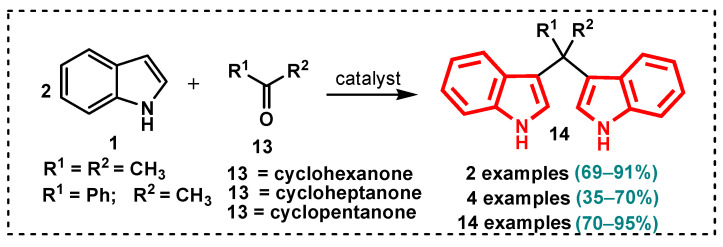
Synthesis of bis(indolyl)methanes **14** from indole **1** and ketones [107,108,109].

Yuan et al. (2019) proposed synthesis of bis(indolyl)methanes **14** reaction of indole **1** with ketones **13** using various catalysts, Amberlyst-15 [107], Silzic (ZnCl_2_/SiO_2_) [108], scandium triflate Sc(OTf)_3_, and *p*-toluenesulfonic acid (*p*-TSA) [109] (Scheme 5). The method’s gentle reaction conditions, straightforward processing, and generally high yields make it advantageous.

### 2.6. Synthesis of Bisindole Derivatives by Reaction of Indoles with Electron-Deficient Alkenes in Aqueous Media

**Scheme 6:**
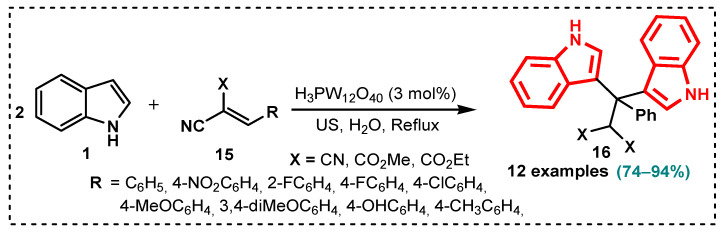
Synthesis of bis(indolyl)methanes **16** from indole **1** and electron-deficient alkenes **15**.

Rahimi et al. (2015) developed a facile approach to the synthesis of bisindole derivatives **16** by the reaction of indole **1** with electron-deficient alkenes **15** (Scheme 6) catalyzed by 12-tungstophosphoric acid as a highly stable, efficient and readily available catalyst under silent and ultrasound irradiation conditions. The benefits of this process are low reaction temperatures, quick reaction times, excellent yields, and the avoidance of hazardous solvents and catalysts [110].

### 2.7. Synthesis of Bis(2-phenyl-1H-indol-3-yl)methanes Through a Domino Gold (I) Chloride Catalyzed Cycloisomerization of 1-Methyl-2-(phenylethynyl)benzene

**Scheme 7:**
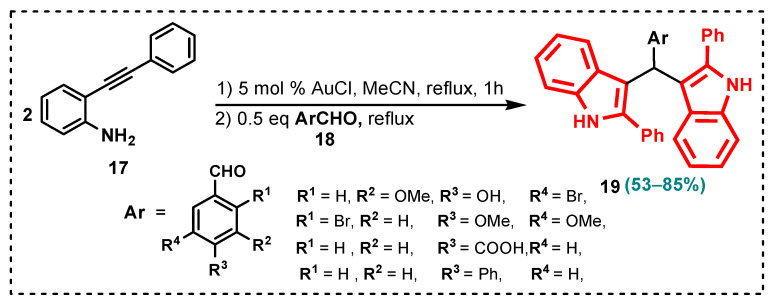
Synthesis of bis(indolyl)methanes **19** using a domino reaction.

In order to synthesize bis(indolyl)methane **19**, Praveen et al. (2009) described a chemoselective process that uses 2-(phenylethynyl)benzenamine **17**, aldehyde **18**, and 5 mol% of AuCl in acetonitrile under reflux (Scheme 7). The reaction proceeded smoothly, with a wide range of functionalized aldehydes, including those containing ether, phenol, carboxylic acid, and polyaromatic and heteroaromatic groups [111].

### 2.8. Synthesis of Homo-bis(indolyl)methanes via Indole-3-alkoxides In Situ Generated Indole-3-alkoxides

**Scheme 8:**
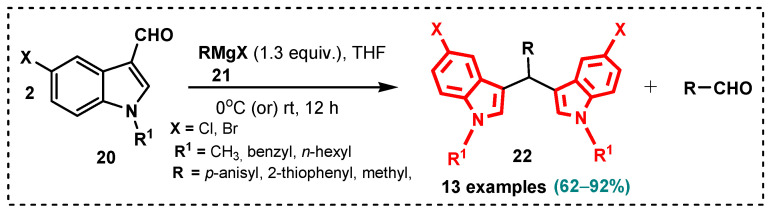
Synthesis of *homo*-bisindolylmethanes **22** using RMgX **21** as reactant.

**Scheme 9:**
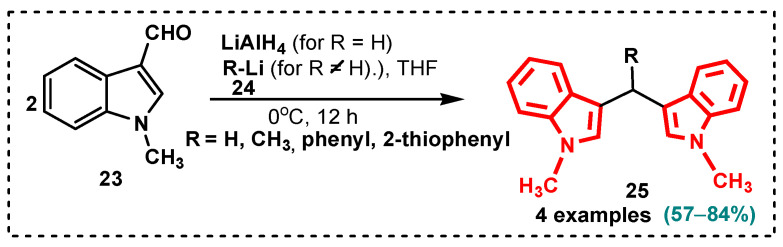
Synthesis of *homo*-bis(indolyl)methanes **25** using RLi **24** as reactant.

**Scheme 10:**
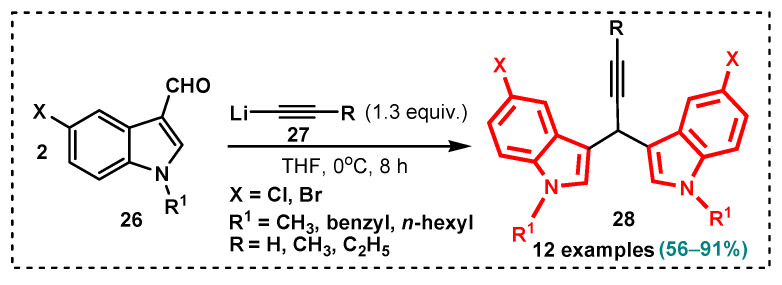
Synthesis of *homo*-bis(indolylmethanes) **28** using alkynyl lithium reagents **27** as reactants.

Chinta and Baire (2016) developed synthesis of *homo*-bis(indolyl)methanes **22** and **25** via indole-3-alkoxides in situ generated from RMgX **21** (Scheme 8) or RLi **24** (Scheme 9) [112]. Also, they carried out a study of various alkynyl lithium reagents **27** as well as aldehyde substrates **26** (Scheme 10) at 0 °C. All the aldehydes and alkynyl lithiums smoothly underwent the cascade reaction and gave *homo*-bis(indolyl)methanes **28** in varying amounts (56–91% yields).

### 2.9. Synthesis of 1-Hydroxyiminomethyl-bis(indolyl) Methanes via Bis-hetero-Diels-Alder Reactions

**Scheme 11:**
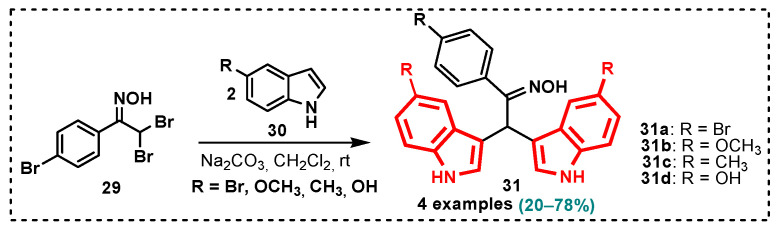
Synthesis of bis(indolyl) oximes **31** via hetero-Diels-Alder reaction.

**Scheme 12:**
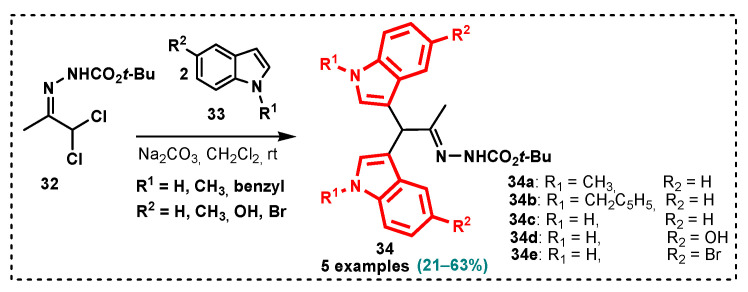
Synthesis of bis(indolyl) hydrazones **34** via hetero-Diels-Alder reaction.

Grosso et al. (2017) reported a one-pot regioselective and stereoselective synthesis of bis(indolyl) oximes **31** (Scheme 11) and bis(indolyl) hydrazones **34** (Scheme 12) via bis-hetero-Diels-Alder reactions of conjugated 2,2-dibromo-1-(4-bromophenyl)ethanone oxime **29** or hydrazone **32** with indoles **30** and **33**. Compounds **31** showed anti-tumoral activity, in particular against lymphoma cell lines [113].

### 2.10. Synthesis of Bisindoles via Multicomponent Reactions

**Scheme 13:**
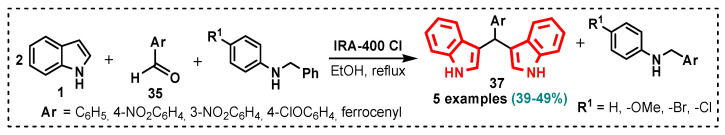
Synthesis of bisindoles **37** using a multicomponent reaction.

Harichandran et al. (2018) reported the synthesis of bisindoles **37** via a multicomponent reaction, with indole **1**, aromatic aldehydes **35**, secondary amines **36**, and Amberlite IRA-400 Cl resin as catalysts (Scheme 13). Although the yields were modest, the method is valuable due to its efficiency and easy handling of the catalyst [114].

### 2.11. Synthesis of Isatin Bisindoles

**Scheme 14:**
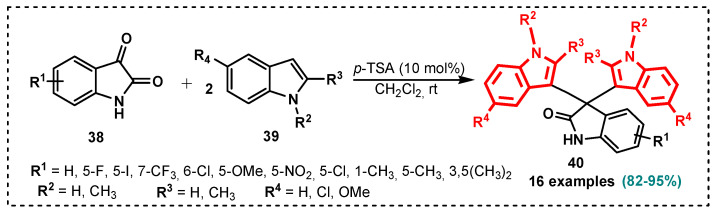
Synthesis of 3,3-bis(1*H*-indol-3-yl)indolin-2-ones **40**.

**Scheme 15:**
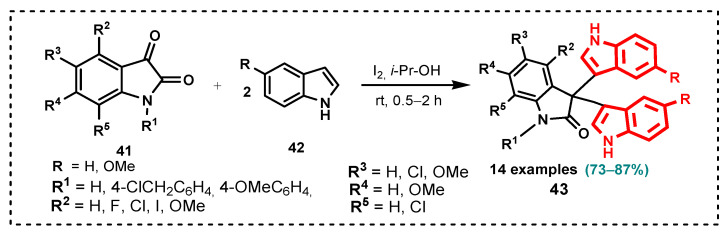
Synthesis of isatin bisindoles **43**.

Yu et al. (2014) reported an efficient synthesis of 3,3-bis(1*H*-indol-3-yl)indolin-2-ones **40** via a reaction of various isatins **38** with indoles **39** in the presence of *p*-TSA in CH_2_Cl_2_ at room temperature (Scheme 14). Also, a series of 2,2-bis(1*H-*indol-3-yl)-2*H*-acenaphthen-1-ones were synthesized the same way [115]. Bonvicini et al. (2022) reported synthesis of isatin bisindoles **43** by the reaction of isatines **41** with indoles **42** in the presence of molecular iodine (Scheme 15) [116].

### 2.12. Synthesis of Acenaphthen Bisindoles

**Scheme 16:**
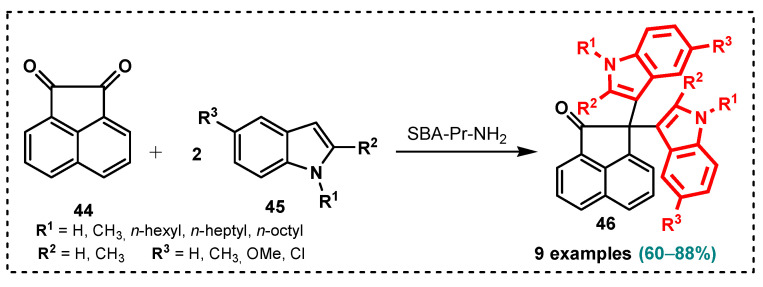
Synthesis of acenaphthene bisindoles **46**.

Ziarani et al. (2015) reported synthesis of 2,2-bis(1*H*-indol-3-yl)-2*H*-acenaphthen-1-ones **46** by condensing acenaphthenequinone **44** and indoles **45** in the presence of a catalytic amount of amino-functionalized silica (SBA-Pr-NH_2_) under solvent-free conditions at 100 °C (Scheme 16). The use of solvent-free conditions makes the process economical and environmentally friendly [117].

**Scheme 17:**
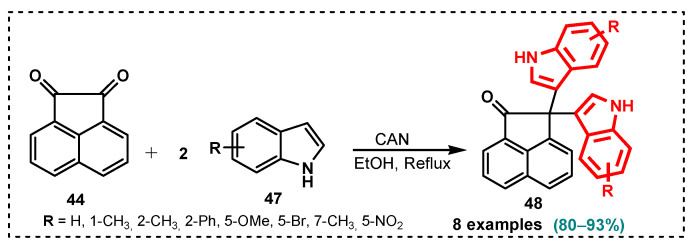
Synthesis of acenaphthene bisindoles **48**.

**Scheme 18:**
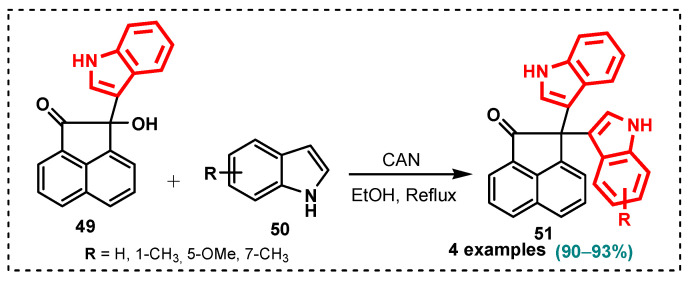
Synthesis of acenaphthene bisindoles **51**.

Feng (2010) reported synthesis of symmetrical 2,2-bis(1*H*-indol-3-yl)acenaphthen- 1(2*H*)-ones **48** in excellent yields using a catalytic amount of ceric ammonium nitrate (CAN) (Scheme 17) as well as synthesis of unsymmetrical 2,2-bis(1*H*-indol-3-yl) acenaphthen-1(2*H*)-ones **51** from the reaction of 2-hydroxy-2-indolylacenaphthen-1(2*H*)-one **49** with indoles **50** (Scheme 18). The author reported that the presence of electron-donating groups on the indole ring led to increased reaction yields, while electron-withdrawing groups, which deactivated the indole ring, had decreased yields [118].

### 2.13. The Reaction Between Amino-Indoles and Acid Chlorides of Dicarboxylic Acids

**Scheme 19:**
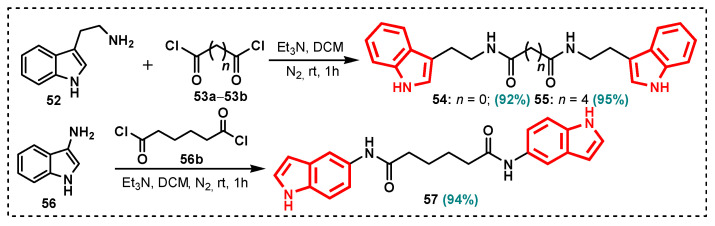
Synthesis of amide bisindoles **54**, **55**, and **57**.

Halawa et al. (2018) reported synthesis of new amide bisindoles **54**, **55**, and **57** by treatment of tryptamine **52** or 5-aminoindole **56** with oxalyl chloride **53a** or adipoyl chloride **53b** (Scheme 19). All bisindoles showed cytotoxic activity against human cervix carcinoma cell line (KB-3-1) [119].

### 2.14. Synthesis of Bisindoles via One-Pot Masuda Borylation–Suzuki Coupling Sequence

**Scheme 20:**
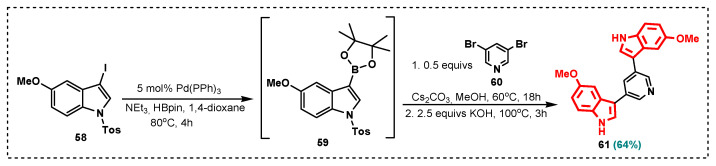
Synthesis of O,O′-dimethyl scalaridine A **62**.

Kruppa et al. (2022) converted 3-iodo-5-methoxy-1-tosyl-1*H*-indole **58** to pinacolyl boronate intermediate **59** by Masuda borylation in the presence of tetrakis(triphenyl phosphane) palladium(0), pinacolyl borane (HBpin), and triethylamine in dry 1,4-dioxane (Scheme 20). After full conversion of the indole **58**, methanol was added to scavenge the remaining excess of HBpin. The subsequent addition of cesium carbonate and half an equivalent of 3,5-dibromopyridine **60** initiated the Suzuki coupling reaction in the same reaction vessel. The addition of potassium hydroxide cleaved the tosyl group, and the alkaloid precursor O,O′-dimethyl scalaridine A **61** was obtained in a yield of 64%. The reaction is important for the synthesis of natural compounds, especially those from marine fauna [120].

## 3. Medicinal Properties of Bisindole Compounds

### 3.1. Bisindolic Compounds with Antimicrobial Properties

Since resistant microbes have emerged as a result of the overuse of antibiotics in many industries in recent decades, researchers are becoming more interested in the synthesis, investigation, and development of novel antimicrobial drugs [121,122]. The subject showed its importance especially in the context of the COVID-19 pandemic, which faced one of the most pressing problems in clinical practice in the context of finding new effective antimicrobial and antiviral compounds against multiresistant pathogens [123,124]. The antibiotic resistance problem is amplified by the ability of bacteria to grow in an adherent state, developing microbial communities called biofilms that exhibit a phenotypic resistance, i.e., tolerance, rendering them 1000-fold more resistant to antibiotic treatments with respect to their planktonic counterparts [125].

**Figure 4:**
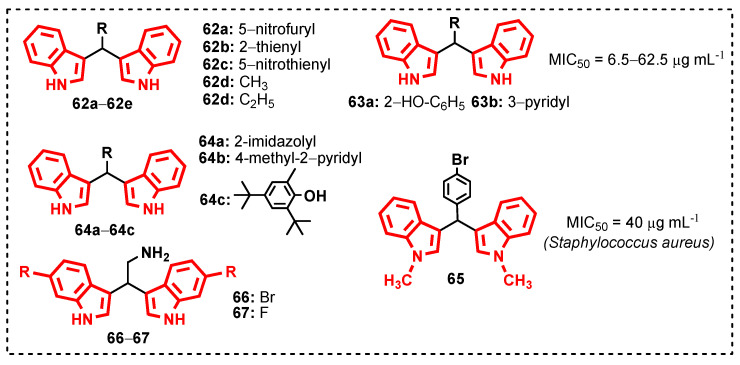
The structures of *bis*(indolyl)methanes **62**–**67** with antimicrobial properties.

**Figure 5:**
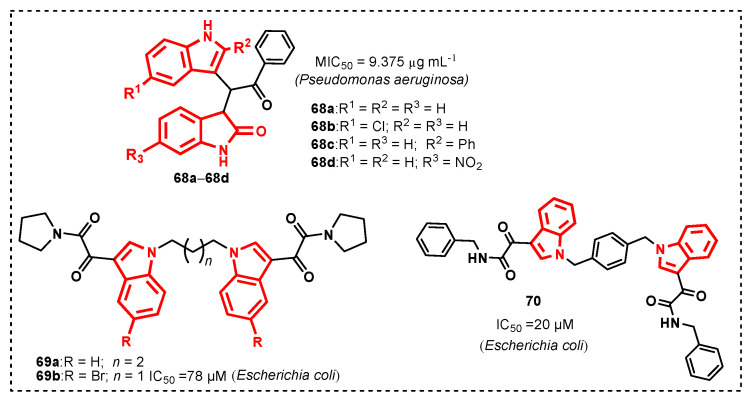
The structures of *bis*(indolyl)methanes **68**–**70** with antimicrobial properties.

**Scheme 21:**
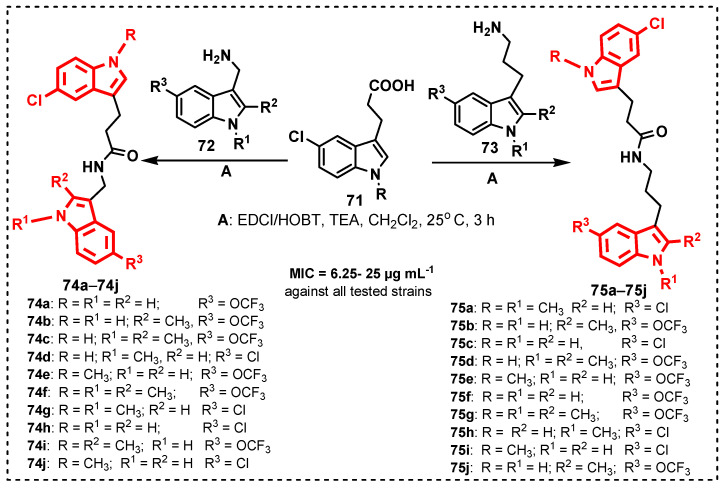
Synthesis of bisindoles **74** and **75**.

Kamal et al. (2009) synthesized a series of bisindoles **62** in good yields (75–96%), using method in Section 2.1 with aluminum triflate (0.5%) as a catalyst in acetonitrile (Figure 4). 5-Nitro heteroaryl containing bisindoles **62a** and **62c** exhibited significant antibacterial activity, and compound **62e** was found to be the most active from this series against all tested strains: *Escherichia coli* MTCC 443, *Pseudomonas aeruginosa* MTCC 1688, *Pseudomonas oleovorans* MTCC 617, and *Klebsiella pneumoniae* MTCC 618 as Gram-negative bacteria and *Bacillus subtilis* MTCC 441 and *Staphylococcus aureus* MTCC 96 as Gram-positive bacteria. Also, compounds **62a**–**62e** demonstrated significant antifungal activity against all tested fungi: *Candida albicans* MTCC 227, *Rhizopus oryzae* MTCC-262, and *Aspergillus niger* MTCC 1344 [126]. Using indium chloride as a catalyst (10 mol%) in isopropanol at 80 °C, Roy et al. (2014) similarly synthesized 3,3′-bis(indolyl)methanes **63a** and **63b**, which showed MIC values of 6.5–62.5 µg mL^−1^ against all tested bacteria: *S. aureus*, *B. subtilis*, *L. lactis*, *Rhodococcus* sp., *A. protophormiae*, *M. smegmatis*, *P. aeroginosa*, *S. flexneri, P. mirabilis*, and *E. coli*. Their higher antibacterial activity in comparison to the other compounds with lower activity was associated with the presence of the -OH group and the 3-pyridyl ring [127]. Beginning with the same reactants, Sarva et al. (2016) synthesized a series of compounds **64** by microwave irradiation in solvent-free conditions. Compounds **64a**–**64c** exhibited the strongest antibacterial activity against a strain of *Staphylococcus aureus.* [128]. Bahe et al. (2020) found that from a series of eight bisindoles, compound **65** had the highest antibacterial activity against *Staphylococcus aureus* with a MIC value of 40 μg mL^−1^ compared to Kanamycin as standard. Bisindole **65** was obtained in the presence of 5.0 mol% *p-*toluene sulfonic acid in acetonitrile at room temperature [129]. Campana et al. (2020) reported similar synthesis for compounds **66** and **67**, and the minimum inhibitory concentration (MIC) of them against the MRSA (*S. aureus* CH 10850) and the MSSA (*S. aureus* ATCC 29213) strains is reported in Table 2, along with their hemolytic activity. Bisindole **67** showed greater disaggregating activity (up to 56% for the examined strains) on preformed biofilm compared to the natural alkaloid **66**. In addition, compound **67** enabled a 256-fold reduction in the MIC of oxacillin for the clinical MRSA strain. The presence of the two halogen atoms, Br and F in compounds **66** and **67,** respectively, was linked to their strong antibacterial activity [130]. The conjugate addition of indole and its derivatives to ene-1,4-dione derived from isatin and acetophenone in the presence of a catalytic amount of molecular iodine in acetonitrile under mild conditions gave 3-(1-(1*H*-indol-3-yl)-2-oxo-2-phenylethyl)indolin-2-one derivatives **68** in good yields (75–90%) (Figure 5). All compounds showed modest antibacterial activity against *Bacillus subtilis* MTCC 441, *Staphylococcus aureus* MTCC 96, *Staphylococcus epidermidis*, *Klebsiella pneumoniae* MTCC 618, and *Escherichia coli* MTCC 443 strains of 75–150 µg mL^−1^ and a very good antibacterial activity against the strain *Pseudomonas aeruginosa* MTCC 741 of 9.375 µg mL^−1^ [131]. Compounds **69** were obtained by Singh et al. (2011) from 2-(1*H*-Indol/5-bromo-1*H*-indol -3-yl)-2-oxoacetyl chloride in two steps: reaction with various amines in presence of K_2_CO_3_ in acetonitrile and 0.5 equivalent of alkane dibromides in presence of NaH in dry acetonitrile at 0–5° C in 16–30% yields. Bisindole **70** resulted from the reaction in the second step of the intermediate *i.* with α,α’-dibromo-*p*-xylene using NaH as base. Compounds **69a** and **70** with 4 mm higher zone inhibition than the solvent control (DMSO + acetone) against *Candida albicans* and **69b** and **70** with IC_50_ of 78 µM and 20 µM, respectively, against *Escherichia coli* were found to be the most promising antifungal and antibacterial candidates [132]. By treating 1-substituted indole-3-carboxylic acid **71** with one of the indolic amines **72** or **73**, two series of bisindoles, **74a**–**74j** and **75a**–**75j** (Scheme 21), resulted. All compounds had good antimicrobial activity, as seen in Table 3. The investigation of antibacterial screening revealed that all the newly synthesized compounds showed moderate to good inhibition at 6.25–25 µg mL^−1^ in DMSO compared to Ciprofloxacin as standard. Compounds **74a**, **74e**, **74h**, **75b**, **75f**, **75i**, and **75j** showed good activity against *E. coli* and *P. aeruginosa*, and compounds **74b**, **74e**, **74j**, **75b**, **75e**, and **75j** showed good activity against *S. aureus* and *S. pyogenes.* Compounds **74a**, **74e**, **74h**, **74i**, **74j**, **75b**, **75f**, **75i**, and **75j** showed good activity against *K. pneumoniae*. Antifungal activity of bisindoles **74** and **75** against all tested fungi, *A. flavus* NCIM 524, *A. fumigatus* NCIM 902, *P. marneffei* recultured, *T. mentagrophytes* recultured, and *Candida albicans* was also good, with values in the range of 6.25–25 µg mL^−1^ compared to ciclopiroxolamine.

**Figure 6:**
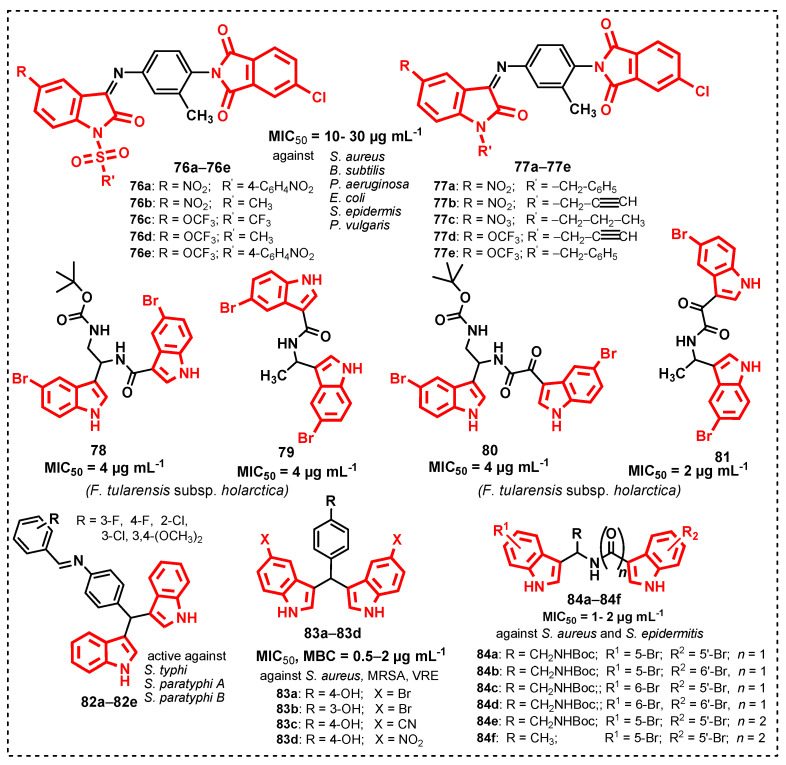
The structures of bisindoles **76**–**84** with antimicrobial properties.

**Figure 7:**
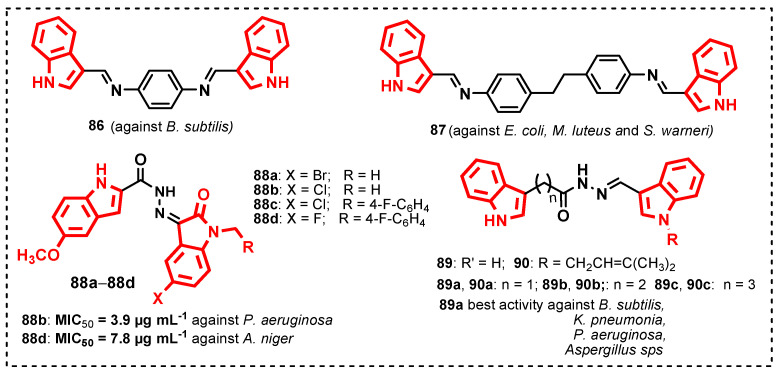
The structures of bisindoles **86**–**87** with antimicrobial properties.

**Scheme 22:**
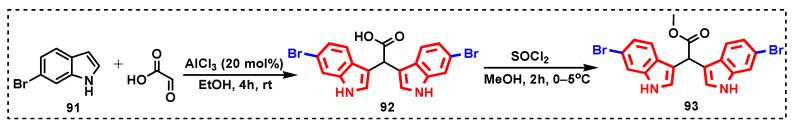
Synthesis of antimicrobial bisindoles **93**.

SAR studies showed that in compounds **74** and **75**, the presence of the halogen and the trifluoromethyl group improved their lipophilicity, while the presence of the amide group was the essential element for hydrogen bonding with the receptor [133]. Kumari and Singh (2013) reported the classic synthesis of two series of bisindole Schiff bases **76** and **77** in good yields (71–77%) (Figure 6). All compounds showed good antibacterial activities against all strains tested*, S. aureus*, *B. subtilis*, *P. aeruginosa*, *E. coli*, *S. epidermis*, and *P. vulgaris*, with a MIC of 10–30 µg mL^−1^. The compounds were often shown to be more potent against Gram-negative bacteria than Gram-positive bacteria according to SAR studies. Furthermore, these Schiff bases’ better antibacterial action was linked to the presence of hydrogen donor-acceptor sites and electron-withdrawing groups [134]. Caspar et al. (2014) reported bisindole compounds **79**–**81** active against *Francisella tularensis* subsp. *holarctica*, with MIC_50_ of 4 µg mL^−1^ for compounds **79**–**80** and 2 µg mL^−1^ for compound **81**. According to SAR investigations, compounds **79**–**81**′s strong antibacterial properties were associated with the presence of bromine at the indole ring’s “5” position [135]. Imran et al. (2014) reported synthesis of a series of bisindole Schiff bases **82** by condensation of 4-(di(1*H*-indol-3-yl) methyl) benzenamine with substituted benzaldehydes. Compounds **82a**–**82e** specifically inhibited *Salmonella typhi, S. paratyphi A*, and *Salmonella paratyphi B* bacterial strains [136]. Sharma et al. (2014) reported synthesis of bis(indolyl)methanes **83**, using different substituted indoles and carbonyl compounds (ketones and aldehydes) in a solvent-free reaction at 90 °C for 10–30 min in good yields (81–96%). Antimicrobial screening of all compounds showed bisindoles **83a**–**83d** were the most potent inhibitors, exhibiting MIC as well as MBC values equal to or less than those of Ciprofloxacin, of 0.5–2 µg mL^−1^ against *Staphylococcus aureus*, MRSA (Methicillin-Resistant *Staphylococcus aureus*), and VRE (Vancomycin-resistant *Enterococcus*). Additionally, SAR investigations demonstrated a strong association between the presence of Br, CN, and NO_2_ groups in the indole’s “5” position and the OH group in the molecule’s “4” phenyl nucleus and outstanding antibacterial properties [137]. Caspar et al. (2015) reported synthesis of a new series of 24 bisindole amides **84** in good yields (40–96%). Minimum inhibitory concentrations (MICs) ranged from 1 to 32 µg mL^−1^ for these compounds against all tested strains, *S. aureus* ATCC 25923, *S. aureus* ATCC 29213, *S. aureus* ATCC 9144, *S. aureus* ATCC 6538, *S. aureus* CIP 65.6, *S. aureus* CIP 103428, *S. aureus* CIP 65.25 (MRSA), *S. aureus* ATCC 33592 (MRSA), *S. aureus* ATCC 106414 (VISA), *S. aureus* SA-1199B (FQR), *S. epidermidis* ATCC 12228, *S. epidermidis* CIP 81.55, and *S. epidermidis* CIP 103627 (MRSE), the most active being **84a**–**84f**, with MICs ranging from 1 to 2 µg mL^−1^. For the three compounds **84a**, **84e**, and **84f**, one MSSA, one MRSA, and 41 CoNS (Coagulase-negative staphylococci) strains belonging to 12 different species were tested. All these strains were susceptible to **84a**, **84e**, and **84f**, with MICs of 1–4 µg mL^−1^. The lowered antibacterial properties of certain compounds 84 were found to be associated with a high hydrophobicity index and high plasma protein binding according to studies of the structure–activity relationship (SAR) [138]. Halawa et al. (2017) synthesized via microwave irradiation nine bisindole derivatives based on *p*-phenylenediamine and 4,4′-ethylenedianiline in very good yields (80–98%). Their antibacterial activity against Gram-positive bacteria *Bacillus subtilis*, *Micrococcus luteus*, and *Staphylococcus warneri* as well as Gram-negative bacteria *Escherichia coli* and *Pseudomonas agarici* were screened by the agar diffusion method and compared to Gentamycin as positive control and dimethyl sulfoxide (DMSO) as negative control. Compound **86** showed the best antibacterial activity against all strains tested, delivering inhibition diameters of 7–14 mm, with the best activity achieved against *B. subtilis*, and compound **87** exhibited moderate activity against *E. coli, M. luteus*, and *S. warneri*, with inhibition diameters of 7–8 mm (Figure 7) [139]. Almutairi et al. (2018) synthesized in good yields (60–94%) nine indole-isatin hybrids **88** by the reaction between 5-methoxy-1*H*-indole-2-carbohydrazide and the appropriate isatin derivative in absolute ethyl alcohol and a catalytic amount of glacial acetic. All compounds were screened for their antimicrobial activity using DIZ (diameter of inhibition zone) and MIC assays. Compound **88b** was the most active congener against *P. aeruginosa* with a MIC_50_ value of 3.9 µg mL^−1^, compound **88d** was the most active congener against *A. niger* (MIC_50_ = 7.8 µg mL^−1^), and compounds **88a** and **88c** were the most active and equipotent against *P. notatum* with a MIC value of 7.8 µg mL^−1^ DFT analysis showed that the good antibacterial properties of bisindoles **88a**–**88b** are positively correlated with the presence of halogen atoms F, Cl, and Br in position “5” of the indole ring [140]. By condensing indole-3-substituted acylhydrazides with 5-substituted-1*H*-indole-3-carbaldehyde in the presence of EtOH and a catalytic amount of glacial acetic acid under refluxing conditions for 20 to 45 min, Choppara et al. (2019) produced a series of N’-((5-substituted-1*H*-indol-3-yl)methylene)-*n*-(1*H*-indol-3-yl) alkanehydrazides **89**. Similarly, a series of N′-((5-substituted-1-(3-methylbut-2-enyl) -1*H*indol-3-yl)methylene)-*n*-(1*H*-indol-3-yl) acetohydrazide **90** was obtained. Compound **89a** had good antimicrobial activity against *B. subtilis*, *K. pneumonia*, *P. aeruginosa*, and *Aspergillus sp*s, compound **89c** had good antibacterial activity against *E. coli*, and compound **90c** had good antifungal activity against *C. albicans* [141]. Holland et al. (2024) synthesized compound **93** from 6-bromo-1*H*-indole **91** in two steps: the reaction with 2-oxoacetic acid on AlCl_3_ (20 mol%) catalyst in EtOH to afford 2,2-bis(6-bromo-1*H*-indol-3-yl)acetic acid **92** and the reaction of **92** with thionyl chloride in MeOH for 2 h at 0–5 °C to obtain methyl 2,2-bis(6-bromo-1*H*-indol-3-yl)acetate **93** (Scheme 22). The MIC_50_ promising values of MSSA (Methicillin-susceptible *Staphylococcus aureus*) ATCC 25923 and MRSA (Methicillin-resistant *Staphylococcus aureus*) ATCC 43300 activities obtained for bisindole acetate **93**, of 8.7 µM and 17.3 µM, respectively, confirmed the proposed cheminformatics predictions in relation to MIA (marine indole alkaloids) chemical similarity analyses coupled with reported bioactivity. The chemical predictions made by MIA chemical similarity analysis were validated by the encouraging MSSA and MRSA activity discovered for 6-dibrominated bisindole **93**. Furthermore, the SAR analysis made it abundantly evident that dibrominated molecule **93** and brominated bisindoles were both preferred over non-brominated ones [142].

**Figure 8:**
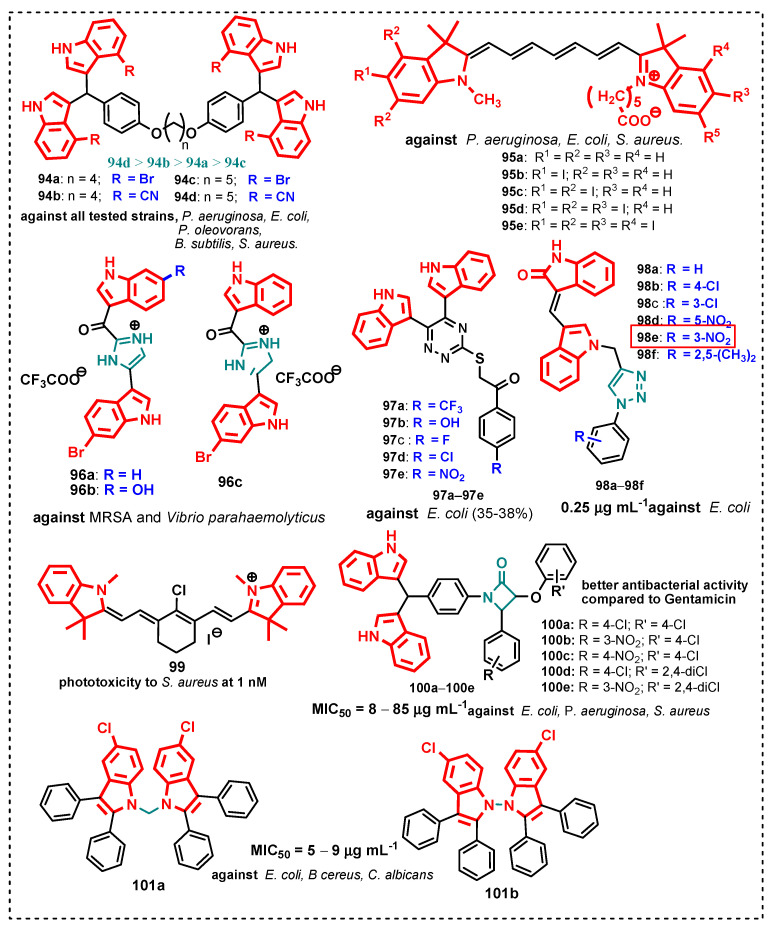
The structures of bisindole compounds **94**–**98** with antimicrobial properties.

**Figure 9:**
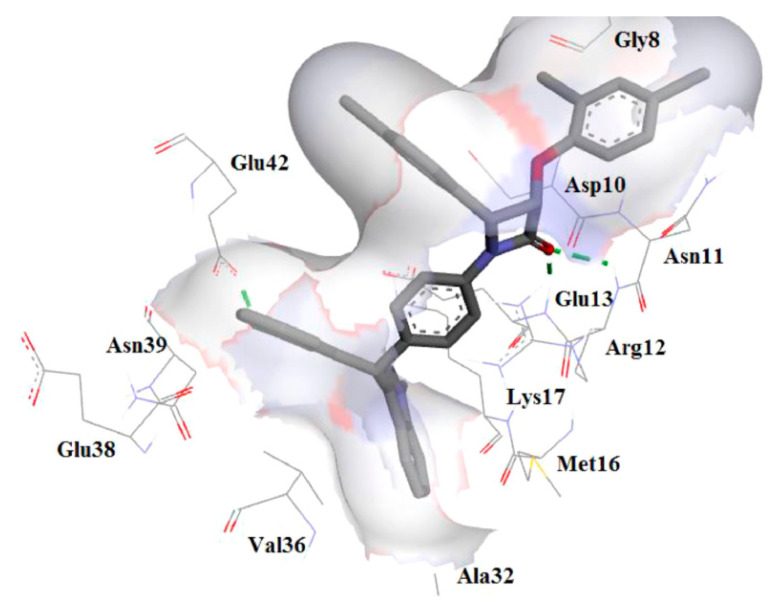
Binding mode of compound **100e** and active site residue in isoform 1 of Bcl-2. Adapted from [149].

Soltani et al. (2020) reported synthesis of bis(indolyl)methane derivatives **94a**–**94d** by condensation of 4,4′-(butane-1,4-diylbis(oxy)) dibenzaldehyde with indole under solvent-free conditions in the presence of various quantities of benzylsulfamic acid at various temperatures (Figure 8). The results showed that antimicrobial activity decreased in the order **94d** > **94b** > **94a** > **94c** against all tested strains, *Pseudomonas aeruginosa* MTCC 1688, *Escherichia coli* MTCC 443, *Pseudomonas oleovorans* MTCC 617, *Staphylococcus aureus* MTCC 96, and *Bacillus subtilis* MTCC 441, compared to Streptomycin as standard. The SAR studies revealed that the presence of nitrile group in compounds **94d** and **94b** contributed to a superior antimicrobial activity compared to compounds **94a** and **94c** (Figure 8) [143]. Semenova et al. (2021) synthesized a series of iodinated heptamethine bisindole dyes **95** containing carboxylic function and reported the photodynamic eradication of Gram-positive (*Staphylococcus aureus*) and Gram-negative (*Escherichia coli*, *Pseudomonas aeruginosa*) microbial pathogens. According to studies, the effectiveness of uncharged, zwitterionic cyanides in photoeradicating *S. aureus* increases as the number of iodine atoms grows up to two; stays about constant for dyes that are two-, three-, and four-iodinated; and decreases when hexaiodinated cyanine is used. Additionally, the mono-iodinated dye had the strongest phototoxic effect on *P. aeruginosa* and *E. coli.* Furthermore, a triethylammonium group adds an extra positive charge that enhances the inactivation of *P. aeruginosa* and *E. coli* while decreasing the photokilling of *S. aureus.* According to SAR research, the carboxylic function may allow these dyes to bind to a variety of carriers in the future [144]. The bisindoles **96** isolated by Kahn et al. (2022) from the sponge *Spongosorites calcicola* were bactericidal to MRSA and gastroenteric pathogen *Vibrio parahaemolyticus*. These compounds were synergistic in combination with conventional antibiotics. Antimicrobial activity of bisindole alkaloids **96** was due to rapid disruption and permeabilization of the bacterial cell membrane [145]. Khan et al. (2023) synthesized in three steps twelve bisindole-based triazine derivatives **97**, starting from 1,2-di(1*H*-indol-2-yl)ethane-1,2-dione. Five of the synthesized compounds, **97a**–**97e**, showed good antibacterial activity against *E. coli* with an inhibition of 35–38% compared with the inhibitory profile of the standard drug Streptomycin (40.33%) [146]. Mokariya et al. (2023) synthesized bisindole-1,2,3-triazole hybrids **98** in very good yields (90–96%) via Cu-catalysed azide-alkyne cycloaddition click reaction. The antimicrobial activity of the compounds was examined against three Gram-negative (*Escherichia coli, Pseudomonas aeruginosa*, *Klebsiella pneumonia*), three Gram-positive (*Bacillus subtilis*, *Staphylococcus aureus*, *Streptococcus pyogenes*), and two fungal strains (*Candida albicans*, *Aspergillus niger*). Compound **98e** was shown to be the best antibacterial agent with a MIC of 25 μg mL^−1^ against *E. coli* among all hybrids **98**. In the SAR analysis, the presence of the Cl atom and NO_2_ group was linked to compounds **98**’s enhanced antibacterial activity. The interactions between compound **98e** and the DNA gyrase active site were discovered by the molecular docking investigation. According to the SwissADME webserver study, most synthetic bisindoles **98** follow the guidelines for drug-likeness [147]. Bisindole dye **99**, which is utilized in numerous assays and imaging applications, has a strong phototoxicity to *S. aureus*, even at 1 nM and low light doses of 33 J⋅cm^−2^, according to Prakash et al. (2023). The dark toxicity of compound **99** against *E. coli* at 10 μM, however, can significantly impact biomedical research that uses this dye [148]. Ranjabari et al. (2023) synthesized in four steps, from indole and 4-nitrobenzaldehyde, ten β-lactam bisindoles **100**. Bisindoles **100a**–**100e** showed the best antibacterial activity against tested strains (*E. coli* ATCC 25922, *P. aeruginosa* 9027, *S. aureus* ATCC 25923) among all tested compounds, even better than the reference Gentamycin. In SAR studies, it was discovered that the nitro group or the chlorine atom at the “4” position of the benzene ring was necessary for their biological activity. The biological activity is more improved when the substituent is present at the “4” position than when it is present at the “2” or “3” positions. Molecular docking studies confirmed the binding modes of these compounds into enzymes and showed the interactions of the active molecules with the main residues to be favorable. Also, molecular docking experiments revealed that bisindole-β-lactam derivatives **100** may inhibit Bcl-2 protein via a hydrogen bond and hydrophobic interactions. Thus, compound **100e** forms a hydrogen bond between the NH of the indole ring and the oxygen of the carbonyl group of Asp35 (NH…O, 2.91 Å) in isoform 1 and two hydrogen bonds with Glu13 and Arg12 in isoform 2 (Figure 9) [149]. Sofan et al. (2023) reported the synthesis of bisindoles **101a** and **101b** and their antimicrobial activity against *Bacillus cereus* ATCC6633, *Staphylococcus aureus* ATCC25923, and *Candida albicans* ATCC10231, compared to the standard drug Penicillin G. Compounds **101a** and **101b**’s decreased and inhibited antibacterial activity (MIC_50_ = 5–9 µg mL^−1^) may be related to the steric hindrance of these bulky molecules. Bisindole **101a** was more efficient against Gram-positive bacteria because of its less steric hindrance than compound **101b**.

The decreased antifungal efficacy of compound **101b** against *Candida albicans* as well as the moderate efficacy of compound **101a** (6 ± 0.14 µg mL^−1^) in comparison to the drug Fluconazole (16 ± 0.06 µg mL^−1^) may also be caused by the same steric hindrance effect [150]. A series of in silico-designed bisindolylglyoxylamide derivatives were verified by Lipinski’s rules and ADMET criteria through successive refinements using three DFT optimization schemes [151].

### 3.2. Bisindolic Compounds with Antitubercular Properties

The high mortality due to tuberculosis, of 1 million deaths annually, led the scientific community to look for new treatment remedies; however, finding new effective antituberculosis compounds requires an extremely long treatment program [152]. Some data from the literature regarding antituberculous bisindoles are presented in the following.

**Figure 10:**
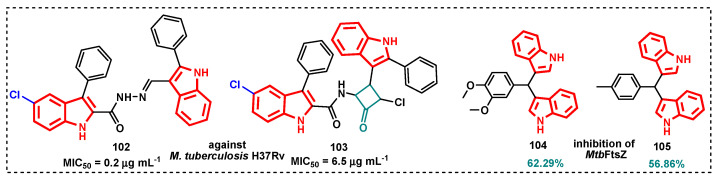
The structures of bisindole compounds **102**–**105** with antitubercular properties.

Walmik and Saundane (2014) reported the synthesis of bisindole **102** by refluxing between 5-chloro-3-phenyl-1*H*-indole-2-carbohydrazide and 2-phenyl-1*H*-indole-3- carbaldehyde using a catalytic amount of acetic acid in methanol (Figure 10). Compound **102** on cyclocondensation with chloroacetyl chloride and a catalytic amount of triethyl amine in dry benzene under reflux conditions afforded 5-chloro-*N*-(3-chloro-2-oxo-4- (2-phenyl-1*H*-indol-3-yl)cyclobutyl)-3-phenyl-1*H*-indole-2-carboxamide **103**. Both compounds had a very good antitubercular activity against *Mycobacterium tuberculosis* H37Rv, the first having MIC_50_ of 0.2 µg mL^−1^ and the second MIC of 6.5 µg mL^−1^ compared to Streptomycin with MIC_50_ of 6.5 µg mL^−1^ [153]. Khadkikar et al. (2018) reported for the first time an efficient and facile green synthesis under ultrasonic irradiation using ammonium molybdate as the catalyst in solvent water of a series of 20 bis(indolyl)methanes as potential *Mycobacterium tuberculosis* (*Mtb*) filamenting temperature-sensitive protein Z (FtsZ) inhibitors. *Mtb*FtsZ inhibition studies of these bisindoles indicated that compounds **104** and **105** showed good inhibition of *Mtb*FtsZ, of 62.26% and 56.86%, and they increased the length of *M. smegmatis* and *B. subtilis* by two folds. Compound **104** inhibited *Mtb* growth with a MIC of 37.5 μg mL^−1^. The authors also carried out detailed molecular docking studies to support the biological activity. Molecular docking calculations using Glide revealed that bisindoles **104** and **105** showed interaction with Asp184, Arg140, Glu136, and Phe180 residues, which are essential for antitubercular activity [154].

### 3.3. Bisindolic Compounds with Antimalarial Properties

Malaria, an infectious disease caused predominantly by *Plasmodium falciparum*, which is widespread in Asia, Africa, and Latin America, is treated with specific drugs depending on the type and severity of the disease, but most therapies applied nowadays are from decades ago. Therefore, there is a need to discover new antimalarial drugs and to establish new strategies to treat this parasitic disease that causes hundreds of thousands of deaths annually [155].

**Figure 11:**
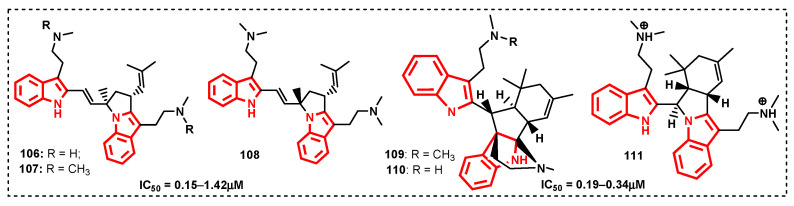
The structures of bisindole compounds **106**–**111** with antimalarial properties.

Fernandez et al. (2008) and Vallakati et al. (2012) isolated from the Australian plant *Flindersia acuminata* Flinderoles A–C **106**–**108** with good antimalarial activities, having IC_50_ values between 0.08 and 1.42 μM against the Dd2 (chloroquine-resistant) *P. falciparum* strain, which they selectivity assessed using the HEK-293 mammalian cell line [156,157]. Robertson et al. (2017) isolated from *Flindersia pimenteliana* bisindoles **109**, **110**, and **111** with IC_50_ (Dd2) antiplasmodial activities against chloroquine-resistant *Plasmodium falciparum* strains (Dd2) of 0.34 ± 0.11, 0.22 ± 0.13, and 0.19 ± 0.04 μM, respectively [158] (Figure 11).

### 3.4. Bisindolic Compounds with Antileishmanial Properties

**Scheme 23:**
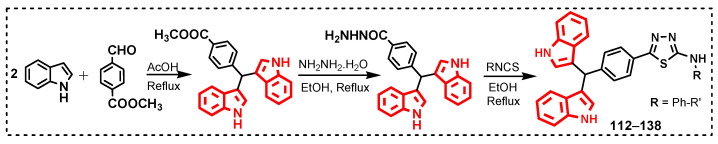
Synthesis of antileishmanial bisindoles **112**–**138**.

**Figure 12:**
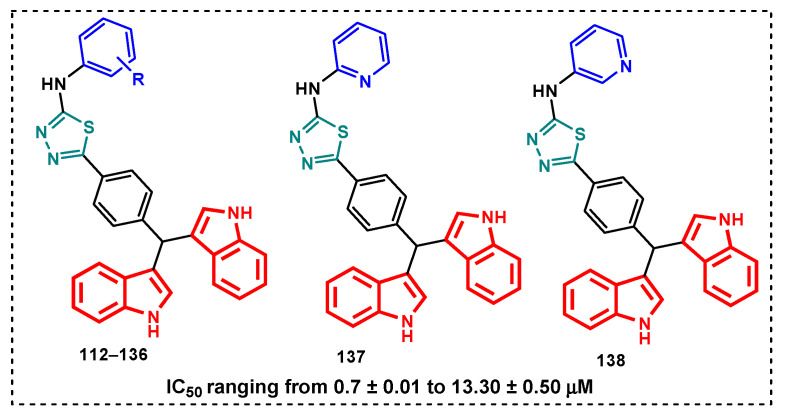
The structures of bisindole compounds **112**–1**38** with antileishmanial properties.

**Figure 13:**
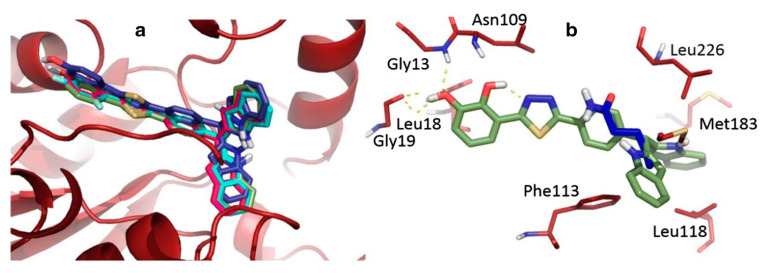
(**a**) Binding mode of the most active compounds in pteridine reductase active site. (**b**) Binding mode of compound **119** (green color) in comparison with pentamidine (blue color). Adapted from [161].

**Scheme 24:**
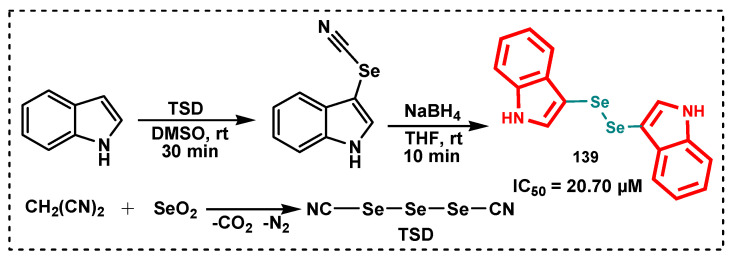
Synthesis of antileishmanial seleno-bisindole **139**.

Leishmaniasis is caused by protozoan parasites of the genus Leishmania and is one of 13 neglected tropical parasitic diseases designated by the World Health Organization’s Tropical Disease Research Program. It has a profound impact on the lives of 12 million individuals globally, primarily affecting the world’s most impoverished populations [159]. In the field of antileishmanial chemotherapy, the importance of *N-*heterocyclic compounds, and particularly of bisindolic compounds, is evident, as seen in the results of numerous studies [160]. Taha et al. (2019) synthesized a series of 27 bisindole analogues **112–138** and evaluated them for their antileishmanial potential. The synthesis of the compounds was performed in three steps: synthesis of methyl 4-(di(1*H*-indol-3-yl) methyl)benzoate using the method mentioned in 2.1., followed by transformation of it to 4-(di(1*H*-indol-3-yl) methyl)benzohydrazide by treatment with hydrazine hydrate in ethanol for 3–4 h at 78 °C, and finally, synthesis of *N*-substituted 5-(4-(di(1*H*-indol -3-yl)methyl)phenyl)-1,3,4-thiadiazol-2-amine by reaction of the second intermediate with the corresponding isothiocyanate in ethanol (Scheme 23, Figure 12). All bisindoles showed excellent inhibitory potential with IC_50_ values ranging from 0.7 ± 0.01 to 13.30 ± 0.50 μM when compared with standard Pentamidine with IC_50_ value of 7.20 ± 0.20 μM (Table 4). Molecular docking studies of these bisindole derivatives on pteridine reductase (PTR) shows that all the active compounds tend to adopt a similar binding mode, as depicted in Figure 13. Comparison of the binding mode of the most active compound **119** with the standard pentamidine used in the study showed that bisindole **119** interacts with the key residues of the PTR active site, establishing hydrophilic and hydrophobic contacts, while pentamidine interacts with fewer hydrophobic residues. This shows that these synthetic bisindoles could be potential candidates as therapeutics against leishmaniasis. According to SAR studies, the antileishmanial activity is enhanced to varying degrees by the presence of certain substituents on the phenyl nucleus. The following substituents are among the most effective: OH, di(OH)_2_, NO_2_, F, Cl, and OCH_3_. Additionally, where they occur on the phenyl ring is crucial. In contrast to other sites, it appears that their presence in the phenyl ring’s “2” position significantly increases the biological activity [161]. Filho et al. (2023) reported synthesis of 1,2-di(1*H*-indol-3-yl) diselane **139** in two steps, as seen in Scheme 24. The study showed that seleno-bisindole **139** has good antileishmanial activity, with an IC_50_ value of 20.70 µM, and can act by interfering with membrane integrity, which leads to mitochondrial dysfunction and the production of ROS (reactive oxygen species) [162].

### 3.5. Bisindolic Compounds with Antiviral Properties

Viral infections are a real threat to each organism at any age, depending on the extent of the disease and the victim [163]. Moreover, in the context of the recent SARS-CoV pandemic and its current consequences, it is necessary to find new antiviral compounds to meet the increasingly diverse requirements on a global level. Indole compounds, and more recently, bisindole ones, have proven to be good antiviral agents, which is reflected in the research we present below [164].

**Scheme 25:**
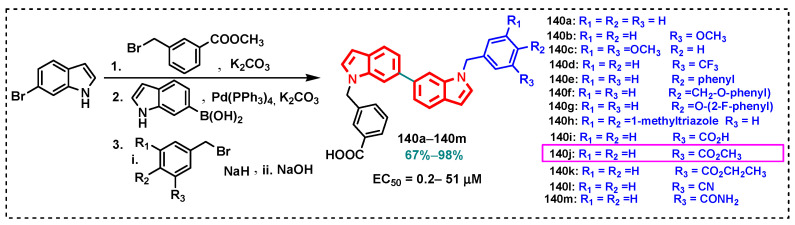
Synthesis of anti-HIV-1 bisindoles **140a**–**140m**.

**Figure 14:**
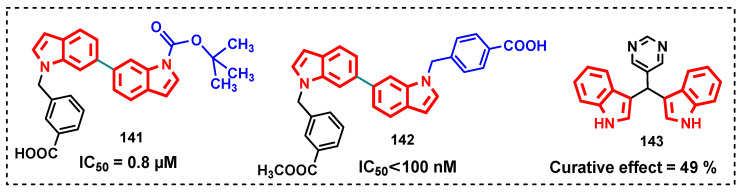
Bisindoles with antiviral properties.

**Figure 15:**
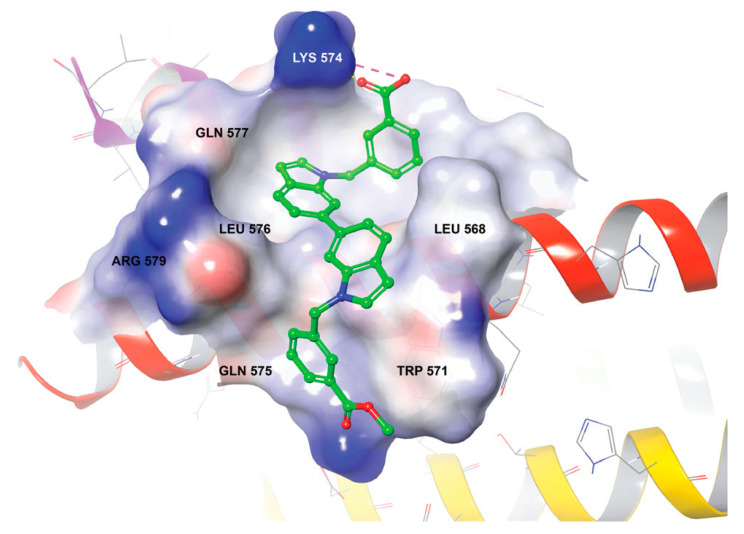
Docked pose of **142** in the hydrophobic pocket of gp41 (PDB 2xra), allowing movement of side chains of Gln 575 and Trp 571. A salt bridge from one carbonyl oxygen on the ligand to Lys574-εNH_2_ is shown as an orange dotted line, and a hydrogen bond is predicted from the second carbonyl oxygen to the lysine eNH_2_. Adapted from [167].

Zhou et al. (2014) reported synthesis in three steps of a series of bisindoles **140** (Scheme 25), which were evaluated for their potential to inhibit HIV-1 in binding, cell fusion, and viral replication assays. The most active bisindole, **140j**, exhibited a binding affinity with K_I_ of 0.6 μM and EC_50_ of 0.2 μM in cell−cell fusion inhibition and viral replication inhibition. Bisindoles **140**’s SAR investigations showed that in order to enter into the hydrophobic pocket, a certain shape, bound conformation, charge, and hydrophobic contact surface were needed. Improved binding affinity, viral replication, and cell–cell fusion inhibition were the outcomes of optimization. The most active molecule, **140j,** had submicromolar activity [165]. In another study, Zhou et al. (2019) reported molecule **141** with promising anti-HIV-1 activity, a value IC_50_ of 0.8 µM, a molecular weight < 500 da, and reduced logP (6.8) compared to previous compounds (Figure 14). The study demonstrated how a small compound intervened in glycoprotein 41’s (gp41) hydrophobic pocket. SubmM’s inhibitory effect against HIV-1 fusion was shown to require both a strong hydrophobic character (logP ≈ 7) and a bisindole core of molecule **141**. Enhancing hydrophobicity did not increase potency over the mid-nM threshold. The hydrogen bonds between the Boc carbonyl group and the Gln 575 pocket, which have the ability to increase specificity and drug affinity, may be the cause of bisindole 141’s superior antiviral activity when compared to other three-ring compounds [166]. Also, Zhou’s group (2021) reported that bisindole **142** is an inhibitor of HIV-1 fusion with an IC_50_ < 100 nM and its interaction with a hydrophobic pocket of glycoprotein-41 (Figure 15). Docking studies suggested an orientation of **142** in the hydrophobic pocket in which one indole group and one benzoic acid moiety fit into the deepest part of the pocket, and the second indole and benzoate extended along a groove bordered by Leu, Gln, and Trp residues, with possible π–π interactions with Trp571 (Figure 15). To accommodate side chain flexibility, especially along the groove, docking was performed using an induced fit model in which Gln 575 and Trp 571 side chains were allowed to move. A large number of non-polar interactions were identified between **142** and residues forming the pocket, and both a hydrogen bond and a salt bridge were predicted between the ligand carboxylic acid and Lys-574 ε-NH_2_. In Figure 15, the different colors on the protein refer to the different charges, positive or negative, highlighted in blue and red, and the amino acids involved in the interaction with the pharmacophore molecule **142** are specified [167]. Gao et al. (2023) reported the synthesis and antiviral activity against tobacco mosaic virus (TMV) of bisindole **143** by the classical method mentioned in point 2.1, with a yield of 71%. Compound **143** exerted a superior inhibitory effect in comparison to the commercial antiviral agent Ribavirin, and therefore it could become a novel antiviral candidate. Molecular docking research and molecular biology experiments found that the potential target of compound **143** was the TMV coat protein [168].

### 3.6. Bisindole Compounds with Anticancer Properties

**Figure 16:**
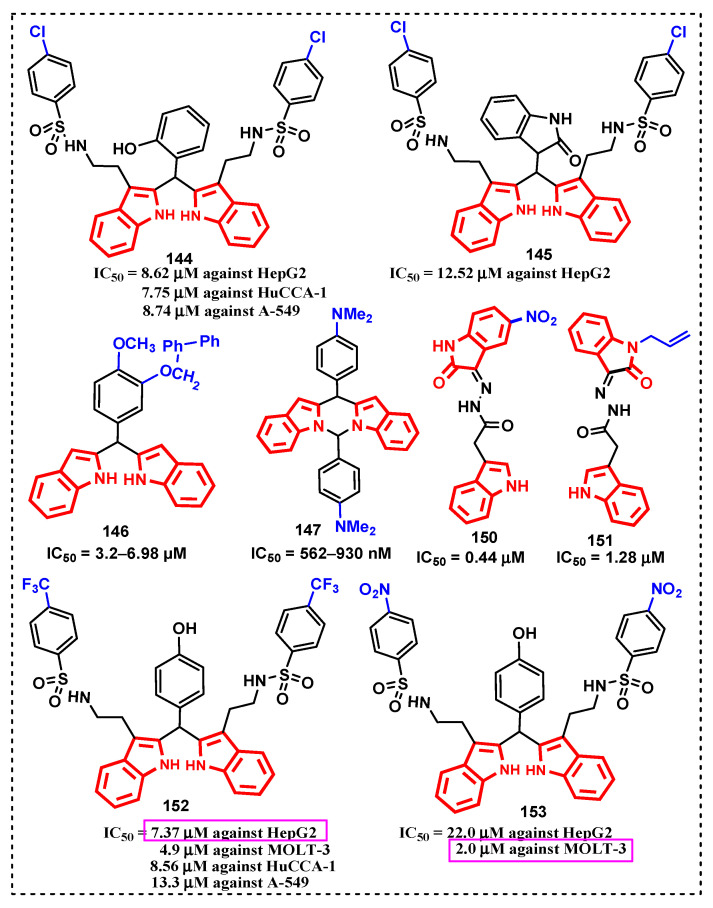
Bisindoles with anticancer properties.

**Scheme 26:**
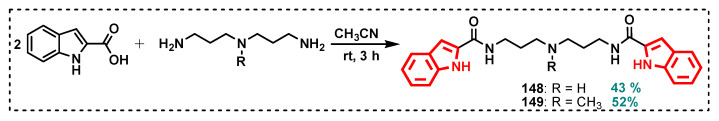
Synthesis of anticancer bisindoles **148** and **149**.

**Figure 17:**
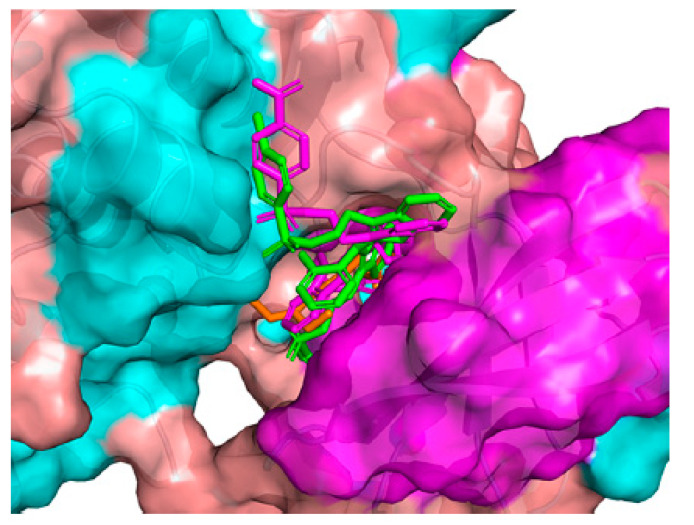
Possible binding mode of the most potent compounds and target proteins. Binding modes of the cocrystallized ligand Ibrutinib (orange), compound **152** (green), and **153** (magenta) against the anticancer target EGFR (PDB ID: 5YU9). Adapted from [178].

**Figure 18:**
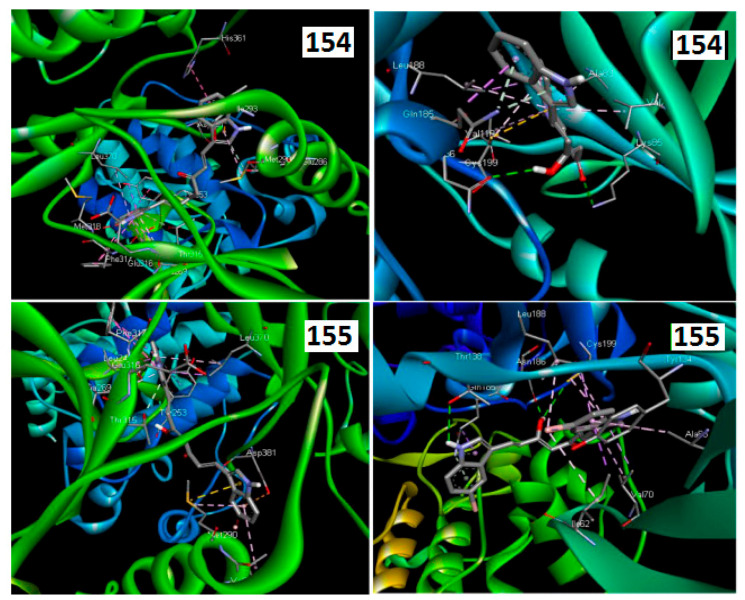
Dock pose of indole derivatives **154** and **155** with Bcr-Abl and GSK-3β proteins. Adapted from [179].

**Scheme 27:**
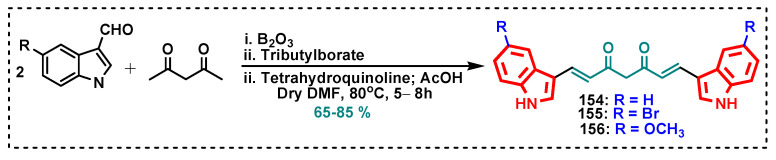
Synthesis of anticancer bisindoles **154**–**156**.

Cancer refers to a series of diseases that occur through uncontrolled cell growth and cell division, when the cells of the body evolve after undergoing successive endogenous and extrinsic mutational processes that lead to abnormal cell proliferation [169]. The number of people with cancer is extremely high; therefore, the World Health Organization (WHO) estimates a number of 29.5 million new cancer cases reported by 2040 [170]. As a result of these literature data, one of the major directions of research is the synthesis of compounds with anticancer properties, and among them, nitrogen heterocyclic compounds occupy a central place due to their remarkable properties, low toxicity, and very extensive therapeutic applications on several types of cancers [171,172,173]. The many kinds of bisindolic anticancer compounds that have been described in the recent literature are therefore presented in more detail. A series of bisindole sulfonamide derivatives were synthesized using the classic reaction (see Section 2.1) and evaluated for their cytotoxic activity by Pingaew et al. (2013). Among all the compounds, the chloro bisindole **144** and trisindole **145** provided 3-fold and 2-fold stronger activity, respectively, against HepG2 cells than Etoposide, with IC_50_ of 8.62 ± 1.04 and 12.52 ± 0.58, respectively, compared to 30.92 ± 5.35 μM (Figure 16). Moreover, chloro bisindole **144** was the most potent compound against HuCCA-1 and A549 cell lines, affording IC_50_ of 7.75 ± 0.37 and 8.74 ± 0.79 μM, respectively. *Para* substitution (R = Cl) on benzenesulfonamide in compounds **144** and **145** had a higher inhibitory potency than analogues with *o-, m-,* and *p-*nitro groups, according to SAR studies [174]. El Sayed et al. (2015) synthesized a series of bisindoles by the classic reaction, in high yields of 86–98% (see Section 2.1). The bisindoles **146** and **147** showed the best anticancer activities of all six tested compounds. Bisindole **146** exhibited good anticancer activities against leukemia cancer cell MOLT-4 (IC_50_ = 6.44 µM); non-small lung cancer cell NCIeH460 (IC_50_ = 5.35 µM); colon cancer cells HCT-116 and HT29 with IC_50_ values of 5.05 µM and 4.74 µM, respectively; melanoma cancer cell M14 (IC_50_ = 3.91 µM); ovarian cancer cell IGROV1 (IC_50_ = 6.98 µM); and renal cancer cells (CAKI-1 and UO-31) with IC_50_ values of 4.78 µM and 3.2 µM. Compound **147** showed the highest antipoliferative activity in a nanomolar scale towards the three selected cancers cell lines; non-small lung cell NCIeH460 with a GI_50_ of 616 nM, ovarian cancer cell line OVCAR-4 with a GI_50_ of 562 nM, and breast cancer cell line MCF7 with a GI50 of 930 nM. The methoxy group’s presence in the *para* position (compound **146**) is significantly more efficient than its presence in the *meta* or *ortho* positions, according to SAR studies. Furthermore, the methoxy and benzyloxy groups in compound **146** primarily contribute to its enhanced activity. While its activities in renal cancer cells showed distinct anticancer activities comparable to compound **146**, the basic substituent NMe_2_ (compound **147**) proved unfavorable in terms of overall anticancer activities [175]. Bisindole polyamines **148** and **149** have been synthesized (Scheme 26) by Szumilak et al. (2017). Their cytotoxic activities (IC_50_) against the PC-3 prostate adenocarcinoma cell line, the DU-145 prostate carcinoma cell line, and the MCF-7 mammary gland adenocarcinoma cell line were determined in the range of 15.51–35.72 µM (Table 5); therefore, it has lower anticancer activity compared to Doxorubicin. However, compounds **148** and **149** presented favorable drug-like properties according to Lipinski’s Rule in the preliminary in silico ADMET screening, thus providingerefore, a promising basis for the further development of potential anticancer drugs. In terms of plasma protein binding (PPB), SAR analyses showed that chemicals **148** and **149** were probably highly bound to plasma proteins (%PPB > 90%). Since the pharmacological activity is typically attributed to a free fraction of the drug, this trait is undesirable because it reduces the efficacy of the “active molecule” [176]. Eldehna et al. (2020) synthesized a series of bisindoles as Topsentin and Nortopsentin analogs. The most potent congeners **150** and **151** against MCF-7 cells (IC_50_ = 0.44 ± 0.01 and 1.28 ± 0.04 µM, respectively) induced apoptosis in MCF-7 cells (23.7- and 16.8-fold increase in the total apoptosis percentage), as evidenced by the externalization of the plasma membrane phosphatidylserine detected by the Annexin V-FITC/PI assay [177]. Pingaew et al. (2021) investigated cytotoxic activities of a series of bisindole compounds against four cancer cell lines (HuCCA-1, HepG2, A549, and MOLT-3). The most promising anticancer compounds were noted to be hydroxyl-bearing bisindoles **152** and **153**, containing CF_3_ and NO_2_ groups, respectively, with the best anticancer properties (Figure 16). Molecular docking revealed that the promising anticancer compound **153** could occupy the active site of the EGFR in a similar manner with that of the cocrystallized ligand Ibrutinib (Figure 17). In Figure 17, the different subunits and protomers of the proteins that interact specifically with the studied molecules **152** and **153** are represented in different colors: green, magenta, and light pink [178]. Parthiban et al. (2022) synthesized the curcumin derivatives **154**–**156** through a three-component reaction (Scheme 27). All synthesized compounds exhibited anticancer properties against Hep-2, A549, and HeLa cell lines and also antioxidant properties, as can be seen in Table 6. The profound anti-cancer and antioxidant activity of methoxy-substituted indole **156** become evident. Molecular docking studies also confirmed the anticancer potential of **154** and **155** by showing the binding interaction with Bcr-Abl and GSK-3β proteins (Figure 18). Compound **156** has a high degree of chemical reactivity, as indicated by the smaller energy gap between the HOMO and LUMO in DFT investigations. Consequently, methoxy substituted indole curcumin 156 was found to be a promising compound, and additional clinical research would determine whether or not the compound might be used medicinally [179].

**Figure 19:**
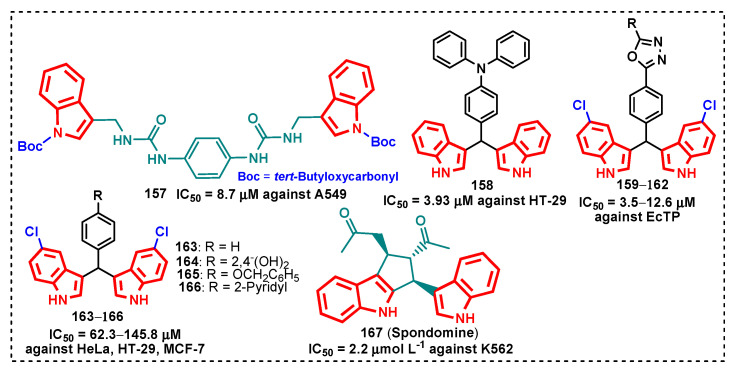
Bisindoles **157**–**162** with anticancer properties.

**Figure 20:**
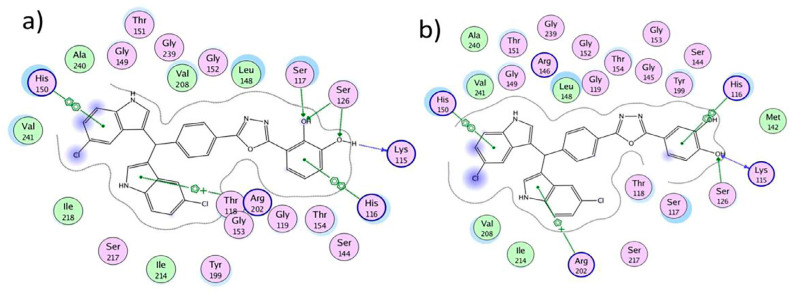
Molecular orbital energy-generated 2D interaction plot of bisindoles (**a**) **161**; (**b**) **162**. Adapted from [160].

Budovska et al. (2023) reported that bisindole **157** significantly inhibited the proliferation of lung cancer cell line A549 (IC_50_ = 8.7 µM) with minimal effects on non-cancer cells. Compound **157** was also shown to induce autophagy by upregulating Beclin-1, LC3A/B, Atg7, AMPK, and ULK1. Furthermore, compound **157** in combination with chloroquine (an autophagy inhibitor) decreased cell proliferation and induced G1 cell cycle arrest and apoptosis. Compound **157** also caused GSH depletion and significantly potentiated the antiproliferative effect of cisplatin (Figure 19) [180]. Gonçalves et al. (2023) reported that the triphenylamine bisindole derivative **158** (see Section 2.1) displayed an IC_50_ of 3.93 µM against the HT-29 cancer cell line. Also, compound **158** showed antiparasitic activity, with an IC_50_ of 3.21 and 3.30 µM against *Trypanosoma brucei* and *Leishmania major*, respectively [86]. Taha et al. (2018) reported that thymidine phosphorylase inhibitors **159**–**162** had an excellent inhibitory effect, i.e., IC_50_ range of 3.5 to 12.6 μM against EcTP (thymidine phosphorylase from *E. coli*), in comparison to the medical standard, 7-Deazaxanthine (Table 7). Docking simulations of compounds **159**–**162** suggest that the optimal inhibitors form interactions with key residues: Lys115, His116, Ser117, His150, Arg202, Val208, Ser217, Ile214, Lys221, and Val241 (Figure 20). The computed GOLD score values (using GOLD docking program 5.4.1) have shown a rational correlation with experimental inhibitory activity of compounds towards thymidine phosphorylase. The chlorine group’s crucial presence in the indole’s “5” position was demonstrated by SAR analyses. Based on the calculated IC_50_ values, Table 7 demonstrates that the 1,3,4-oxadiazole ring has phenyl-OH groups. The presence of 2,4-dihydroxyphenyl is very advantageous, as seen by compound **159**’s IC_50_ of 3.50 ± 0.01 μM. Moreover, significant inhibitory activity was lost upon conversion to -OCH_3_ or removal of one of the -OH groups [92].

**Figure 21:**
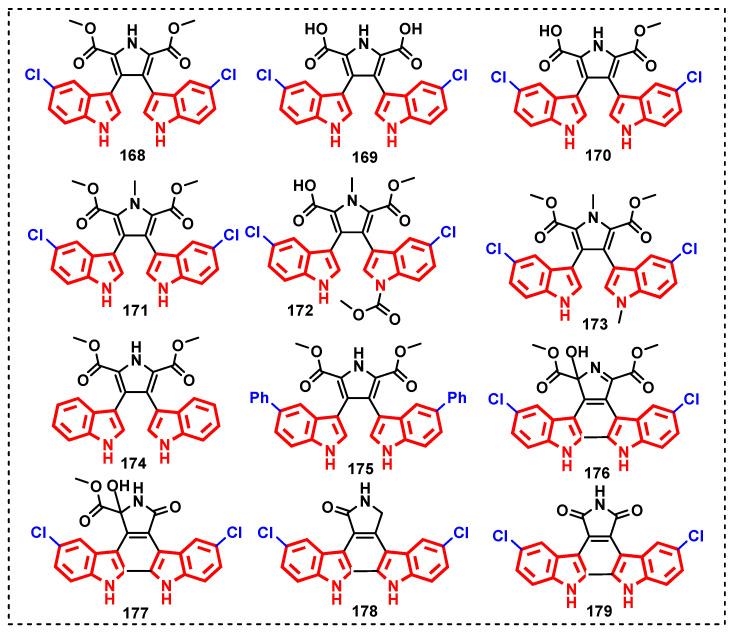
Bisindoles **168**–**179** as MARK4 inhibitors.

**Scheme 28:**
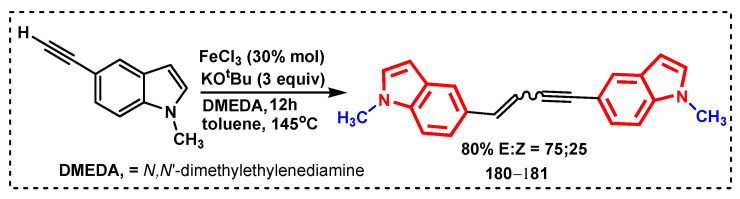
Synthesis of anticancer bisindoles **180**–**181**.

Naidu et al. (2013) reported synthesis of bisindoles **163**–**166** in excellent yields using the electrophilic substitution reaction of indole with aromatic aldehydes in the presence of polymer-supported dichlorophosphate (PEG-OPOCl_2_) as an efficient green catalyst with a short reaction time (2–10 min) at room temperature. Their anticancer properties were evaluated against colon, breast, and cervical cancer cell lines. As shown in Table 8, bisindole **163** showed good anticancer activity on three kinds of cancer cells [94]. Grosso et al. (2017) reported the anticancer activity of bis(indolyl) oximes **31a**–**31d** (Scheme 11) and bis(indolyl) hydrazones **34a**–**34e** (Scheme 12) against three tumoral and one non-tumoral cell lines (S17) (Table 9). The best IC_50_ values for these compounds are highlighted in green in Table 9. Two IC_50_ values determined for the compounds, 3.23 mM for **31c** on the EL4 cell line and 7.65 for **34b** on the non-tumor S17 line, were better than the positive control, etoposide [113]. Jin et al. (2021) reported that the bisindole alkaloid **167** (spondomine) showed excellent cytotoxicity against K562 cell lines with an IC_50_ value of 2.2 μmol L^−1^ (Figure 19) [181]. Voura et al. (2022) reported the synthesis of a series of bisindole alkaloids **168**–**179** as new MARK4 inhibitors (Figure 21). The compounds proved to be MARK4 inhibitors according to enzyme inhibition assays, with IC_50_ values in the low micromolar range (Table 10). Moreover, the experiments showed that bisindoles **168** and **176** induce early to late apoptotic events in A549 cells. In the SAR study, the presence of the chlorine atom in the “5” position of the indole was found to be essential, as well as the COOH and COOCH_3_ substituents in the “2” and “5” positions of the pyrrole ring. The Autodock Vina software in conjunction with PyRx was used to perform docking studies on bisindoles **168**–**179** in order to anticipate molecular interactions with the catalytic region of MARK4 (PDB: 5ES1). With binding affinities ranging from 7.2 to 10.7 kcal mol^−1^, it was found that all compounds were stabilized by a variety of non-covalent interactions, including hydrogen bonds with residues within the MARK4 active site. The pyrrole moiety’s NH group is pointed in the direction of the phosphate pocket, where Glu182 interacts with it. In compounds **169** and **170**, a hydrogen bond was also established with the residue Lys64 [182]. Ranjabari et al. (2023) reported a moderate antiproliferative activity on the MCF-7 tumor line, with IC_50_ values of 12.42–32.30 µM for bisindoles **100a**–**100e** (Figure 8). The inclusion of a xanthene group at the C-3 position of the β-lactam ring increased the anticancer bioactivity according to SAR studies. Cytotoxicity activity was reduced when the *para* position on the phenyl ring substituent in **100c** was substituted for the *meta*-nitro group on **100b**. This is explained by the enzyme’s active site’s incorrect geometric orientation and altered hydrogen bond interactions with the essential amino acids [149]. Midya et al. (2023) reported the iron-catalyzed synthesis of the fluorescent enyne bisindoles **180** and **181** (Scheme 28). The *trans*-enyne **181** investigated for its potential to trigger cell death in cancer cells exhibits cytotoxic effects on HepG2 cancer cells and halted the cell cycle during the G2/M phase [183].

### 3.7. Bisindolic Compounds with Anti-Inflammatory Properties

Medicines with anti-inflammatory properties reduce the inflammation produced by an inflammatory agent. Unlike opioids, which have an impact on the central nervous system, anti-inflammatory drugs represent about half of analgesics, which relieve pain by reducing inflammation [184]. The literature mentions a series of bisindoles with anti-inflammatory properties [185], which are presented below. The therapeutic anti-inflammatory action of these nonsteroidal anti-inflammatory drugs (NSAIDs) is achieved by blocking the synthesis of prostaglandins and selectively inhibiting cyclooxygenase.

**Figure 22:**
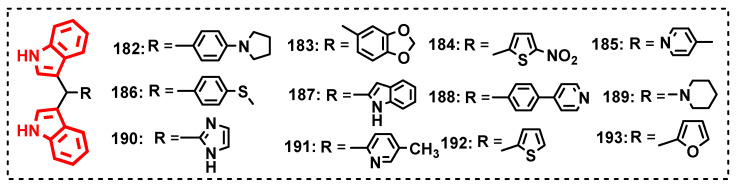
Bisindoles **182**–**193** with anti-inflammatory properties.

**Figure 23:**
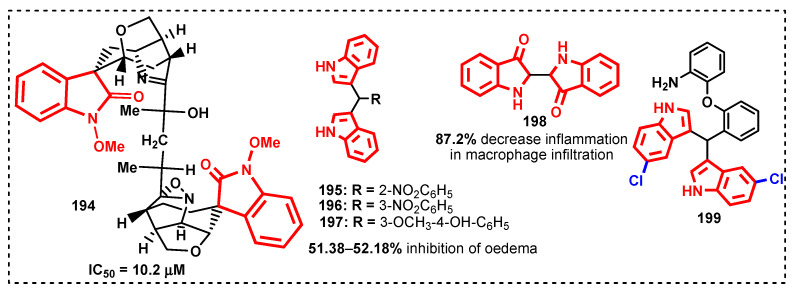
Bisindoles **194**–**197** with anti-inflammatory activity.

Sarva et al. (2016) reported the anti-inflammatory activity of bisindoles **188**–**193** and their significant anti-inflammatory effects at two different concentrations, 50 µg mL^−1^ and 100 µg mL^−1^ (Figure 22). Moreover, bisindoles **188**, **190**, **191**, and **192** exhibited significantly greater anti-inflammatory effects compared to the reference drug Diclofenac (Table 11) [128]. Qu et al. (2013) reported the anti-inflammatory activity of bisindole alkaloid **194** isolated from *Gelsemium elegans*. Bisindole **194** (Geleganimine B) demonstrated anti-inflammatory properties by inhibiting lipopolysaccharide-induced proinflammatory factors in BV2 microglial cells, showing an IC_50_ value of 10.2 μM (Figure 23) [186]. Sujatha et al. (2009) reported bisindoles **195**–**197** with enhanced anti-inflammatory activity (51.38–52.18% inhibition of edema IE) without ulcerogenic liability considering Indometacin (76.66% IE) as the standard [187]. Mizobuti et al. (2022) conducted in vivo studies and morphological analyses of the anti-inflammatory effects of indigo **198** on the dystrophic DIA muscle. The mdxIND group exhibited a significant decrease in inflammatory area (by 36.5%) and macrophage infiltration area (by 87.2%) when compared to the mdx muscle cells [188]. Zhang et al. (2021) showed that treatment with bisindole **199** effectively reduced Iba-1-positive microglia cells in the cerebral cortex and hippocampus after cerebral ischemia. The treatment with **199** significantly improved neuroinflammation, as characterized by Interleukin (IL)-6, tumor necrosis factor (TNF)-α, and oxidative stress, characterized by reactive oxygen species (ROS) and superoxide dismutase (SOD), in comparison to the model group [189].

### 3.8. Bisindolic Compounds with Anti-Alzheimer Properties

**Figure 24:**
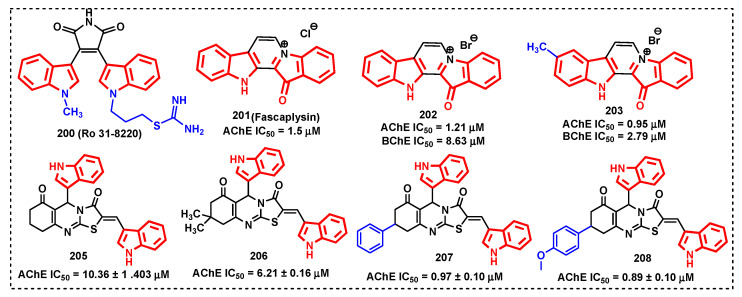
Bisindoles **200**–**197** with anti-Alzheimer properties.

**Scheme 29:**
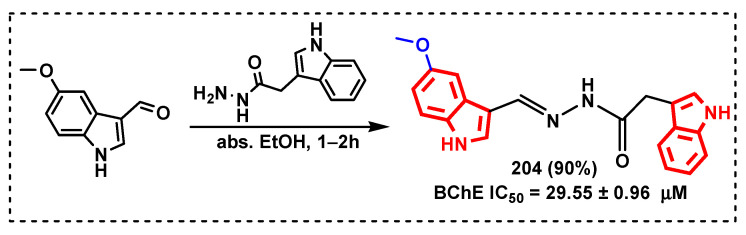
Synthesis of anti-Alzheimer bisindole **204**.

**Figure 25:**
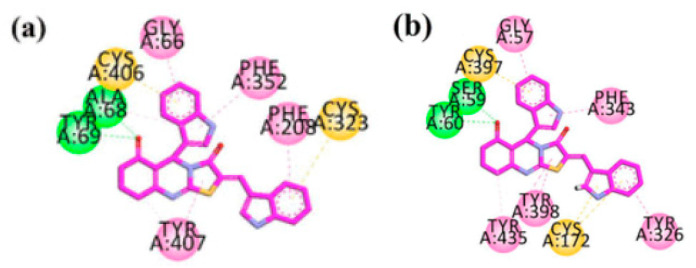
(**a**) A 2D interaction diagram of compound **205** within the binding site of MAO-A (PDB ID = 2Z5X); (**b**) 2D interaction diagram of compound **205** within the binding site of MAO-B (PDB ID = 2V5Z). Adapted from [195].

According to Shim et al. (2019), a small bisindole **200** (Ro 31-8220) restored tau-induced memory impairment, enhanced midge motor functions, and decreased phosphorylated tau species both in vitro and in vivo. Furthermore, **200** decreased tau phosphorylation and PKC activity in a human neuroblastoma cell line, thus being a good inhibitor of tau protein-induced neurotoxicity (Figure 24) [190]. Bharate et al. (2012) reported a bisindolic alkaloid **201** (Fascaplysin) as an AChE inhibitor, with an IC_50_ of 1.5 μM [191], and also as a protector against Aβ oligomer-induced neuronal death. Molecular modeling investigations were carried out to ascertain the conformation, orientation, and interaction of bisindole **201** in the AChE active site. The structure and orientation of **201** in the AChE active site were discovered by docking experiments. The structure of Fascaplysin (**201**) is planar. Upon docking in the AChE active site, it was discovered to occupy the AChE active site, which is a deep and narrow gorge that contains two ligand binding sites: an acylation site (also known as a catalytic anionic site, or CAS) at the gorge’s base and a peripheral site (also known as a peripheral anionic site) close to the gorge entrance [192]. Pan et al. (2019) synthesized bisindoles **202** and **203** as the most potent inhibitors of AChE from the synthesized series, which reduced neurotoxicity in the nanomolar range. By lowering pro-inflammatory cytokine expression and preventing Aβ-induced tau hyperphosphorylation in vivo, compounds **202** and **203** showed improvement in mice’s cognitive impairment brought on by scopolamine or Aβ oligomers [193]. Angelova et al. (2023) synthesized melatonin-containing bisindole **204** by the reaction between 5-methoxy-1*H*-indole-3-carbaldehyde and 2-(1*H*-indol-3-yl)aceto hydrazide in absolute EtOH for 1–2 h (Scheme 29). Compound **204** showed promising butyrylcholinesterase (BChE)-inhibitory activity, with an IC_50_ of 21.12 ± 1.48 µM, better than the control Galantamine with an IC_50_ of 29.55 ± 0.96 µM. Compound **204** was anticipated by an in silico pharmacokinetics analysis to be well absorbed, digested, and eliminated, with the majority of it being able to pass across the blood–brain barrier. Additionally, compound **204** was assessed for blood–brain barrier permeabilities using the parallel artificial membrane permeability test (PAMPA). Future uses of bisindole **204** as a Multi-Target Directed Ligand (MTDL) in complicated disorders such as severe depression and AD that target melatonin MT1 and MT2 receptors as well as AChE and/or BChE enzymes are suggested by docking studies [194]. Nadeem et al. (2022) reported that bisindoles **205**–**208** have AChE inhibition in the range of micromolar concentrations (Figure 24) and also excellent MAO-B inhibition. Bisindole **205** was found to be one of the most potent inhibitors of human MAO-B with an IC_50_ value of 0.13 μM. The methoxy substituent gives compound **208** its enhanced anti-Alzheimer activity, and the docking investigation showed that the presence of heteroatoms on the bisindole core, thiazolone, and pyrimidine rings is crucial for the interaction with the receptor. Docking investigations were conducted on MAO isoforms to assess the binding orientations and interaction patterns of bisindole **205**. Figure 25a displays the 2D interaction plot of bisindole **205** within the binding site of MAO-A. Compound **205** interacts with Phe208, Gly66, Phe352,Cys323, Tyr407, Cys406, and Tyr444. Amino acids Ala68 and Tyr69 engage in hydrogen bond interactions, whereas cysteine residues (Cys323 and Cys406) interact through π−sulfur interactions. The interaction diagram of bisindole **205** within the binding site of MAO-B is displayed in Figure 25b [195,196].

### 3.9. Bisindolic Compounds with Antioxidant Properties

Reactive oxygen species (ROS) and reactive nitrogen species (RNS) are associated with numerous pathologies and conditions, including rheumatoid arthritis, cancer, atherosclerosis, Alzheimer’s disease, Parkinson’s disease, and others. The development of antioxidant molecules that can neutralize ROS and RNS, thereby reducing radical-induced oxidative injury, has been the prudent approach used until now to prevent diseases associated with oxidative stress [197]. We present below a series of bisindoles with antioxidant properties mentioned in the literature.

**Figure 26:**
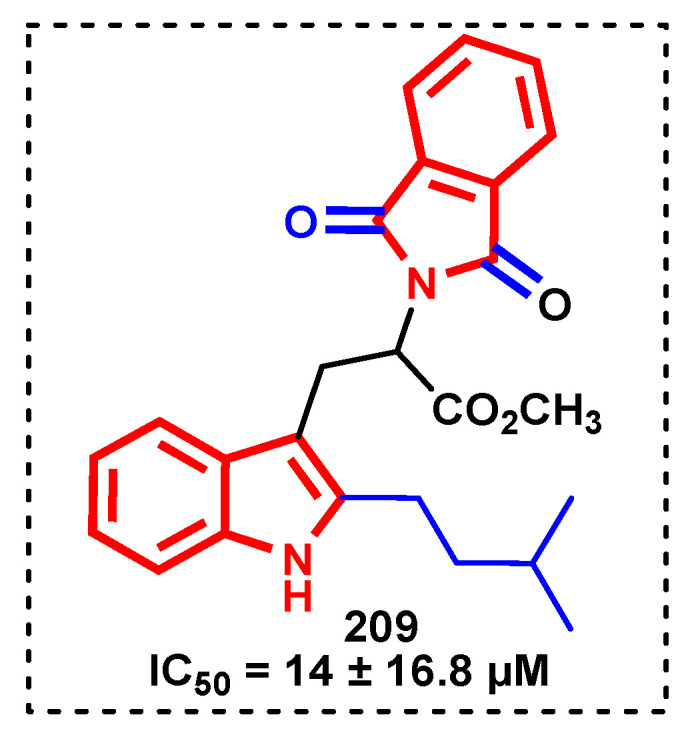
Bisindole **209** with antioxidant properties.

**Scheme 30:**
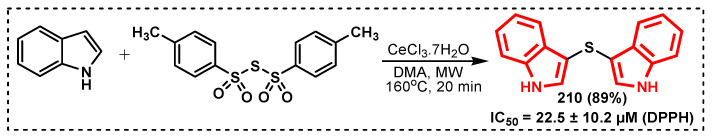
Synthesis of antioxidant bisindole **210**.

Estevão et al. (2010) reported that bisindole **209** has excellent antioxidant activity against peroxynitrite anion (ONOO^−^) in the presence of NaHCO_3_, with an IC_50_ of 14 ± 16.8 µM. This indicates that **209** is an efficient scavenger of RNS and, in addition, this radical stabilization is highly dependent on the type of substituents on the indole moiety and their relative positions (Figure 26) [198]. Silveira et al. (2013) synthesized di(1*H*-indol-3-yl)sulfanes by the reaction of benzimidazole with 4-methylbenzenesulfonic thioanhydride in DMA (dimethylacetamide) under microwave irradiation (160 °C) and Ce(III) as the promoter (1.0 mmol) (Scheme 30). The IC_50_ value (sample concentration required to inhibit 50% of radicals) of 22.5 ± 10.2 µM and maximum inhibition (I_max_) of 89.9 ± 2.5% were found for bisindole **210** in the DPPH assay [199]. Mizobuti et al. (2022) reported the in vitro and in vivo activity of indigo. Comparing the indigo-treated group to the mdx group in in vitro experiments revealed a significant decrease in SOD-2, GPX-1, and GSH levels (36.6%, 61.4%, and 15.1%, respectively). The experimental groups’ levels of GSR and catalase did not differ significantly from one another. Regarding the impact of indigo on the antioxidant defense system in the dystrophic DIA muscle, in vivo studies showed that the mdxIND group significantly reduced the levels of SOD-2 (by 62.1%), CAT (by 42.3%), GPX-1 (by 57.1%), GSR (by 38.6%), and GSH (by 29.3%) in comparison to the mdx group [188]. Ranjabari et al. (2023) reported the antioxidant activity of bisindoles **100a**–**100e** using the DPPH assay and ascorbic acid as the standard molecule. All compounds showed good antioxidant activity, with IC_50_ values in the range of 179–223 µg mL^−1^, as seen in Table 12. Additionally, SAR investigations found that the compounds’ varying levels of antioxidant activity are caused by the C-3 and C-4 substituents of the β-lactam [151].

### 3.10. Bisindole Compounds with Antidiabetic Properties

Diabetes mellitus, characterized by high blood sugar and dyslipidemia, is a chronic disease spread all over the world and that is expanding at an alarming rate. Type 2 diabetes is primarily caused by insulin resistance, although the specific deficiencies in insulin action are still unknown. Neuropathy, nephropathy, retinopathy, and cardiovascular disease are among the long-term complications that patients with diabetes mellitus are most likely to experience [200]. Therefore, it is imperative to find new antidiabetic compounds to meet the continuing demands of this widespread disease. Below, we present some bisindole compounds reported in the literature to have antidiabetic properties.

**Scheme 31:**
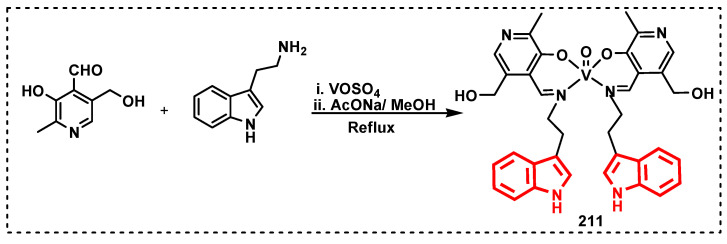
Synthesis of antidiabetic bisindole **211**.

**Figure 27:**
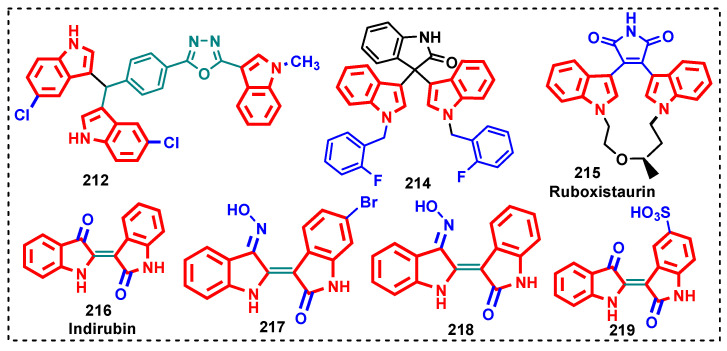
Bisindoles **212**–**219** with antidiabetic properties.

**Scheme 32:**
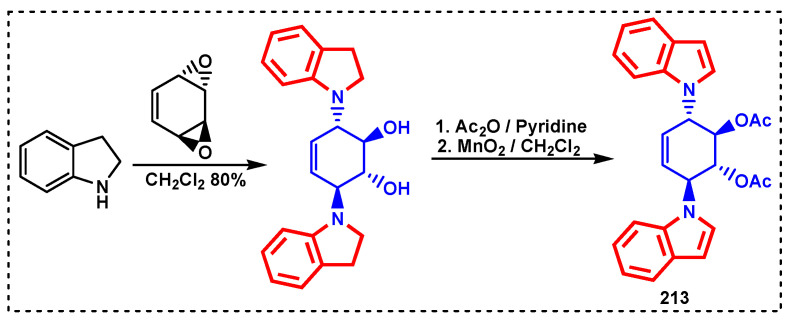
Synthesis of antidiabetic bisindole **213**.

Ki et al. (2016) reported the synthesis of the non-cytotoxic bisindole vanadyl-Schiff base **211** using a one-pot synthetic method as indicated in Scheme 31. According to the trials, **211** has strong anti-inflammatory and insulin-mimetic properties. In particular, bisindole **211** is able to control insulin signaling by deactivating JNK-1 and adding a phosphate group to the tyrosine part in order to activate IRS (insulin receptor substrate) [201]. A new series of oxadiazole-trisindole hybrids was reported by Taha and colleagues (2017). Compound **212** showed the best antidiabetic activity by inhibiting α-glucosidase (Figure 27). In the SAR investigation, the presence of the chlorine atom on bisindole molecule **212** was linked to its higher antidiabetic activity. Docking analyses of the binding mode of compound **212** (IC_50_ = 2.00 ± 0.01 µM) showed that it adopts a binding mode that will fit the entire furrow of the binding site of α-glucuronidas,e and the chloro group of **212** is strongly bound to the active site of α-glucosidase. The high inhibitory activity on α-glucosidase is due to the creation of a powerful hydrogen bond [202]. Cavdar et al. (2012) reported synthesis of cyclohexene-linked bisindole **213** in two steps (Scheme 32). Bisindole **213** exhibited moderate β-glycosidase inhibition assay, with an IC_50_ value of 67.7 µM [203]. Wang et al. (2017) reported a series of 3,3′-bisindole derivatives with inhibitory activity on α-glucosidase. Compound **214**, which contains group 2-fluorobenzyl, showed the best antidiabetic activity, with an IC_50_ value of 5.98 ± 0.11 µM [204]. Mayati et al. (2015) reported a series of bisindolymaleimides, analogs of Staurosporine as Protein Kinase C beta inhibitors (PKC-b1 and b2). Bisindole **215** (Ruboxistaurin) exhibited excellent antidiabetic activity. The same research group reported three series of cyclic indole compounds as orally available selective PKC-b inhibitors [205]. Indirubin **216**, a major component of *Polygonum tinctorium*, synthesized by Sugai et al. (2020), accelerates the differentiation of 3T3-L1 cells by increasing triglyceride accumulation in the cells, thus becoming a good activator of peroxisome proliferator-activated receptor gamma (PPARg), and could be used for the treatment of type 2 diabetes [206,207]. Docking and ADMET prediction revealed that 6-bromoindirubin-3-oxime **217** could be a potential inhibitor of GSK-3 and therefore an antidiabetic compound [208]. Other researchers have identified two more structures, indirubin-3′-monoxyme **218** and indirubin-5-sulfonic acid **219**, as selective inhibitors of GSK-3β [209].

### 3.11. Bisindolic Compounds with Analgesic Properties

The term “analgesics” refers to a group of pharmaceuticals used to manage and treat pain, including opioids, acetaminophen, nonsteroidal anti-inflammatory drugs [210,211], antidepressants, antiepileptics, and local anesthetics [212,213,214,215]. Bisindole compounds are also notable as analgesics. Several structures of bisindoles with analgesic properties are presented below.

**Scheme 33:**
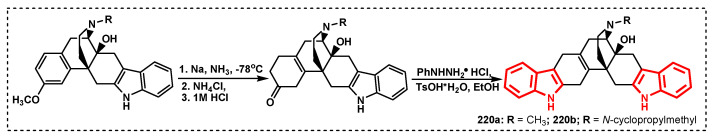
Synthesis of analgesic bisindoles **220a** and **220b**.

Li et al. (2009) synthesized analgesic bisindoles **220a** and **220b** with yields of 60% and 34%, respectively (Scheme 33). *N*-cyclopropylmethyl-bisindole **220b** demonstrated moderate affinity at all three opioid receptors, whereas *N*-Me-bisindole **220a** lost affinity at the d receptor but maintained some affinity at the l receptor. With a K_i_ value of 34 nM, compound **220b** has greater potency at the l receptor and is selective against the d and j receptors by two and four times, respectively. SAR studies evaluated bisindoles **220a** and **220b** for their binding affinity at all three opioid receptors (*µ*, *δ*, and *k*) using a specific procedure. These studies revealed that bisindole **220a** showed partial agonistic stimulation at the *µ* receptor, while its *N*-cyclopropylmethyl-congener **220b** produced good agonistic stimulation at all the three opioid receptors, indicated that it is a universal opioid receptor partial agonist possessing EC50 values of 46–110 nM [216].

### 3.12. Bisindolic Compounds as Carbonic Anhydraze II Inhibitors

The enzymes known as carbonic anhydrases are members of the family that catalyzes the interconversion of carbon dioxide, water, and the dissociated ions of carbonic acid (i.e., hydrogen and bicarbonate ions). They are categorized as metalloenzymes because the majority of carbonic anhydrases have a zinc ion in their active site [217]. These enzymes assist in moving carbon dioxide and preserving the acid–base equilibrium. According to research, human carbonic anhydrase is crucial for many physiological functions, and aberrant enzyme activity is connected to a number of illnesses, including glaucoma, leukemia, and epilepsy [218].

**Figure 28:**
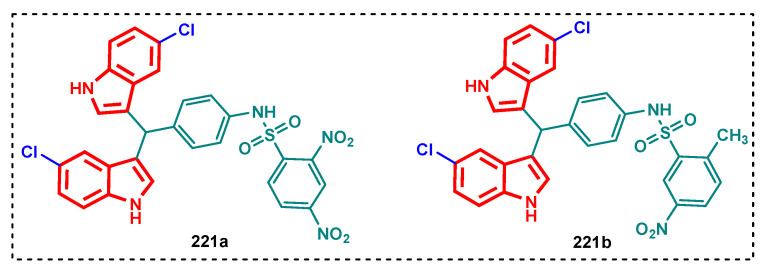
Bisindoles **221a** and **221b** as carbonic anhydrase II inhibitors.

Imran et al. (2016) synthesized 45 sulfonamide-bis(indolyl)methanes using a three-step procedure. Some of the compounds showed carbonic anhydrase II inhibition activity. Thus, pharmacophore studies indicate that the bis(indolyl)methane moiety has a major role in the suppression of carbonic anhydrase II activity. According to docking studies, molecules containing nitro substituents (**221a** and **221b**, Figure 28) can interact with Zn^2+^ ions, Thr199, His94, His96, and His119, interfering with the hydrogen bond network of ZnAOHAThr199AGlu106. Compound **221a**’s bulky nitro substituent at the ortho position (IC_50_ = 15.6 ± 0.79 µM) stops it from interacting with other residues, such as Thr199 and Thr200. Because compound **221b**’s methyl substituent at the ortho position (IC_50_ = 14.3 ± 2.4) reduces the steric hindrance effect, the second oxygen atom of sulfonamide can bind with Thr199 (2.51 Å). Increased stability of the ligand-receptor complex may result from hydrogen bonding between NH on the indole ring and Glu69. The nitro substituent at the *para* position of compound **221a** fits into the core of the active site according to docking studies for active compounds of the sulfonamide class [219].

## 4. Conclusions and Perspectives

In this review, we have tried to review the general and special synthesis methods of bisindoles as well as their medicinal properties, with the results reported so far in the literature. The bisindole core has proven to be an essential part in directing the therapeutic properties of the synthesized compounds. It practically determines the wide range of biological properties presentedŞ antimicrobial, antituberculosis, antileishmanial, antimalarial, anticancer, antioxidant, anti-Alzheimer, anti-inflammatory, antidiabetic, carbonic anhydrase II inhibiting, etc. The need for functionalization of indole nuclei with various functional groups, such as NO_2_, NH_2_, COOH, CONH, halogens, Cl, Br, and I, to obtain compounds with remarkable therapeutic properties has been clearly formulated so far. Furthermore, the significance of functionalizing bisindoles’ indole rings and other benzene or aromatic nuclei has been emphasized by SAR investigations. The docking experiments stated above emphasize how crucial it is to functionalize the indole rings with polar groups in order to create hydrogen bonds between the protein binding site and the pharmacophore, which is the bisindole molecule. The results presented so far in the literature represent an open door to new research, the synthesis of new bisindole compounds, the study of the mechanisms of action, the conduct of several in vivo studies, as well as preliminary research for bringing some of these compounds to the market as drugs for the treatment of various diseases. May this review serve as a springboard for a plethora of studies and a window into the state of affairs in this area.

## Figures and Tables

**Table 1:** Screening of efficiency of catalysts used in the synthesis of bisindoles from indoles and aromatic aldehydes.

Entry	Catalyst	Reaction Conditions	Yield	Reference
1	KHSO_4_	MeOH, rt, 7 h	37–80%	[86]
2	Bi(NO_3_)_3_∙5H_2_O	CH_2_Cl_2_, rt, 20 min	98%	[87]
3	HClO_4_–SiO_2_	MeOH, rt, 1–60 min	88–95%	[88]
4	Ln(OTf)_3_	EtOH, H_2_O, rt, 12–36 h	64–93%	[89]
5	Sm_2_O_3_/SiO_2_	Solvent free, 60–70 °C, 5–60 min	70–96%	[90]
6	Ni nanoparticles	Solvent free, 80 °C, 60–90 min	85–95%	[91]
7	Glacial acetic acid	Reflux, 8 h	88%	[92]
8	Taurine	H_2_O, sonication, 1–24 h	59–90%	[93]
9	Poly(ethylene glycol) (PEG) supported dichlorophosphate (PEG-OP(O)Cl_2_)	rt, 2–10 min	92–95%	[94]
10	Tetramethyl guanidinium chlorosulfonate	CH_2_Cl_2_, rt, 40 min	90–96%	[95]
11	Pentafluorophenylammonium triflate (PFPAT)	CH_3_CN, rt, 5–60 min	60–98%	[96]
12	Aluminumdodecatungstophosphate (AlPW_12_O_40_)	CH_3_CN, rt, 1–25 min	71–98%	[97]
13	1-butyl-3-methylimidazolium tetra-fluoroborate ([bmim]BF_4_)	rt, 3–7.5 h	85–93%	[98]
14	1-butyl-3-methylimidazolium hexafluorophosphate ([bmim]PF_6_)	rt, 4–8 h	78–90%	[98]
15	N,N,N,N-tetramethylguanidinium trifluoroacetate (TMGT)	100 °C, 25–60 min	93–98%	[99]
16	N,N,N,N-tetramethylguanidinium triflate (TMGTf)	100 °C, 3.5–5 h	86–92%	[99]
17	[(*n*-propyl)_2_NH_2_][HSO_4_]	MW, 3–5 min	85–99%	[100]
18	Highly uniform and hyper-cross-linked polyphenanthrene and poly-pyrene microspheres	Neat, 25–100 °C, 1 h	20–96%	[101]
19	Bio-surfactant, chickpea leaf exudates	*i*-Pr-OH, 80 °C, 30 min	96%	[102]
20	Ytterbium(III) triflate/sodium dodecyl sulfate (Yb(OTf)3/SDS)	H_2_O, rt, 1.5 h	84%	[103]

**Table 2:** Antimicrobial activity and hemolytic activity (%) of compounds **66** and **67** [130].

Compound	MIC (µg mL^−1^)		Hemolysis (%)
	*S. aureus* CH 10850 (MRSA)	*S. aureus* ATCC 29213	
**66**	2	2	2.96 ± 0.02
**67**	32	16	2.32 ± 0.06

**Table 3:** Antimicrobial activity of compounds **74a**–**74j** and **75a**–**75j**.

Compounds	MIC in µg/mL				
	*S. aureus* ATCC 25923	*E. coli* ATCC 24922	*P. aeruginosa* ATCC 27853	*K. pneumoniae* Recultured	*S. pyogenes*
**74a**	6.25	6.25	6.25	6.25	12.5
**74b**	6.25	12.5	6.25	25	6.25
**74c**	6.25	12.5	6.25	25	25
**74d**	12.5	12.5	25	12.5	6.25
**74e**	6.25	6.25	6.25	6.25	6.25
**74f**	12.5	12.5	25	12.5	25
**74g**	25	12.5	12.5	12.5	25
**74h**	6.25	6.25	6.25	6.25	12.5
**74i**	25	6.25	25	6.25	12.5
**74j**	6.25	6.25	25	6.25	6.25
**75a**	12.5	25	25	25	25
**75b**	6.25	6.25	6.25	6.25	6.25
**75c**	25	25	25	25	6.25
**75d**	12.5	12.5	25	12.5	6.25
**75e**	6.25	12.5	12.5	12.5	6.25
**75f**	6.25	6.25	6.25	6.25	12.5
**75g**	12.5	12.5	25	12.5	25
**75h**	25	12.5	12.5	12.5	25
**75i**	6.25	6.25	6.25	6.25	12.5
**75j**	6.25	6.25	6.25	6.25	6.25
Ciprofloxacin	6.25	6.25	6.25	1.56	6.25

**Table 4:** Antileishmanial potential of bisindoles **112**–**138**.

Compound	R	IC_50_ (µM ± SE)	Compound	R	IC_50_ (µM ± SE)
**112**	4-OCH_3_	5.3 ± 0.3	**126**	2-F	0.95 ± 0.05
**113**	3-OCH_3_	6.4 ± 0.2	**127**	3-OH-4-OCH_3_	2.30 ± 0.1
**114**	3-NO_2_	4.3 ± 0.2	**128**	3-Cl	9.10 ± 0.3
**115**	3,4-di(OH)_2_	0.80 ± 0.01	**129**	2-OH-5-OCH_3_	2.25 ± 0.20
**116**	4-CH_3_	6.70 ± 0.20	**130**	2-NO_2_	3.30 ± 0.20
**117**	2-CH_3_	5.20 ± 0.2	**131**	2.4-di(OH)_2_	3.30 ± 0.10
**118**	2.5-di(OH)_2_	3.50 ± 0.20	**132**	3-CH_3_	13.30 ± 0.50
**119**	2.3-di(OH)_2_	0.7 ± 0.01	**133**	4-Cl	6.9 ± 0.30
**120**	4-F	1.50 ± 0.05	**134**	2-Cl	3.20 ± 0.1
**121**	2-OH-4-OCH_3_	4.20 ± 0.2	**135**	3-F	6.80 ± 0.2
**122**	3-OH	2.65 ± 0.10	**136**	3-OH	5.8 ± 0.20
**123**	4-NO_2_	6.8 ± 0.20	**137**	2-Pyridyl	1.60 ± 0.1
**124**	3.5-di(OH)_2_	5.80 ± 0.30	**138**	3-Pyridyl	7.60 ± 0.20
**125**	4-OH	3.60 ± 0.20			
Pentamidine	–	7.20 ± 0.20			

SE = standard error.

**Table 5:** Cytotoxicity of bisindole polyamines **148** and **149**.

Compound	IC_50_ (µM)		
	PC-3	DU-145	MCF-7
**148**	27.59	16.46	0.44
**149**	35.72	15.86	1.28
Doxorubicin	1.22	0.58	1.51

**Table 6:** Anticancer and antioxidant activities of indole derivatives **154**–**156**.

No	Compounds	In Vitro Studies			
		Anti-Cancer (IC_50_)			Antioxidant
		Hep-2 (μM)	A549 (μM)	HeLa (μM)	Reduction Level of DPPH (%)
1	**154**	25 ± 0.9	20 ± 1.6	8 ± 0.7	73.41
2	**155**	18 ± 1.3	28 ± 30	6 ± 0.52	81.95
3	**156**	12 ± 0.8	15 ± 1.2	4 ± 0.5	90.50
4	Doxorubicin	10 ± 0.8	0.65 ± 0.04	1 ± 0.09	–
5	Ascorbic acid	–	–	–	99.91

**Table 7:** Inhibitory potential and gold fitness scores of synthesized compounds **159**–**162** against EcTP.

Compound	R	GOLD Score	IC_50_ Value (μM) ± SEM
**159**	2,4-dihydroxyphenyl	73.51	3.50 ± 0.01
**160**	2,5-dihydroxyphenyl	73.10	6.10 ± 0.01
**161**	2,3-dihydroxyphenyl	72.56	9.30 ± 0.10
**162**	3,4-dihydroxyphenyl	70.97	12.16 ± 0.15
7-Deazaxanthine	-	60.65	38.68 ± 1.12

**Table 8:** Anticancer activities of bisindoles **163**–**166** (IC_50_ concentration).

No	Compound	Hela Cells (µM)	HT-29 (µM)	MCF-7 (µM)
1	**163**	86.3	62.3	138
2	**164**	95.4	128.3	145.8
3	**165**	129.5	98.4	>150
4	**166**	139.3	89.7	>150

**Table 9:** Cytotoxicity of compounds **31a**–**31d**, **34a**–**34e**, and etoposide (positive control) expressed as IC_50_ (µM) against three tumoral and one non-tumoral cell lines.

No	Compound	Cell Line			
		EL4	THP-1	U-937	S17
1	**31a**	7.65	12.2	18.6	ND
2	**31b**	9.29	22.6	18.1	17.7
3	**31c**	3.23	6.34	9.79	11.5
4	**31d**	15.6	15.5	12.8	21.9
5	**34a**	9.99	25.3	14.2	ND
6	**34b**	5.56	38.8	NA	7.65
7	**34c**	15.3	30.0	25.0	50.0
8	**34d**	15.4	28.6	156.9	NA
9	**34e**	22.5	36.2	28.3	41.3
10	Etoposide	4.90	1.82	1.10	10.4

ND—not determined; NA—not active.

**Table 10:** IC_50_ (µM) values of the aATPase inhibition assay of MARK4 for compounds **168**–**179**.

Compound	Enzyme Inhibition	Compound	Enzyme Inhibition
**168**	2.88	**169**	3.99
**170**	4.32	**171**	0.47
**172**	1.86	**173**	5.67
**174**	4.83	**175**	0.69
**176**	1.87	**177**	4.97
**178**	3.99	**179**	0.56

**Table 11:** In vitro anti-inflammatory activity of bisindoles **182**–**195**.

Compound	% Hemolysis	% Protection	% Hemolysis	% Protection
	50 ^a^	100 ^a^	50 ^a^	100 ^a^
**182**	69.44	58.33	30.60	41.67
**183**	47.22	30.55	52.78	69.45
**184**	52.77	38.80	47.30	61.20
**185**	55.83	45.55	44.17	54.45
**186**	79.72	67.50	20.28	32.50
**187**	63.88	55.55	36.12	44.45
**188**	3.33	27.77	66.67	72.23
**189**	66.66	52.77	33.34	47.23
**190**	41.60	25.02	58.40	75.00
**191**	30.55	22.22	69.45	77.78
**192**	44.44	27.70	55.56	72.30
**193**	72.22	52.77	27.78	47.23
Diclofenac	75.00	66.60	25.00	33.40

^a^ Concentration in µg mL^−1^.

**Table 12:** Antioxidant activity measured as % scavenging of DPPH radical.

Compound	IC_50_ (µg mL^−1^)
**100a**	211
**100b**	184
**100c**	223
**100d**	197
**100e**	179
Vitamin C	195

## Data Availability

Not applicable.
